# Probing the Stability of Halogenated Carbon Atomic Wires in Electrospun Nanofibers via Raman Spectroscopy

**DOI:** 10.1021/acs.jpcc.5c02960

**Published:** 2025-07-08

**Authors:** Simone Melesi, Piotr Pińkowski, Bartłomiej Pigulski, Nurbey Gulia, Sławomir Szafert, Chiara Bertarelli, Chiara Castiglioni, Carlo S. Casari

**Affiliations:** † Department of Energy, Micro and Nanostructured Materials Laboratory-NanoLab, Energy, 18981 Politecnico di Milano, Via Ponzio 34/3, Milano 20133, Italy; ‡ Faculty of Chemistry, 49572 University of Wrocław, 14 F. Joliot-Curie, Wrocław 50-383, Poland; § Department of Chemistry, Materials and Chemical Engineering “Giulio Natta”, Politecnico di Milano, Piazza Leonardo da Vinci 32, Milano 20133, Italy

## Abstract

Carbon atomic wires (CAWs) are one-dimensional (1D) sp-carbon nanostructures with remarkable electronic, mechanical, and optical properties, but their instability limits their practical applications. Embedding them in solid matrices can enhance their stability. This work reports the first example of electrospun nanofibers embedding halogenated CAWs. A solution of poly­(methyl methacrylate) and CAWs in *N*,*N*-dimethylformamide was electrospun using various parameters to investigate the effects on fiber morphology and diameter. Halogenated CAWs were successfully incorporated with a minimal morphological impact. Raman spectroscopy confirmed effective embedding and CAWs stability during electrospinning. The halogenated CAWs showed resistance to degradation for at least six months and demonstrated enhanced thermal stability when embedded within nanofibers. Additionally, our work investigated the influence of different halogen terminations on the degradation kinetics of CAWs upon exposure to these conditions. Similarly, photodegradation studies revealed improved photostability within fibers and demonstrated how CAWs chemical structure affects degradation pathways, including possible homolytic C–X bond cleavage. This work introduces electrospun nanofibers as a novel platform for stabilizing CAWs, offering advantages over thin films, such as better homogeneity, larger surface area, and comparable stability. These findings open new perspectives for CAWs-based nanocomposites in electronics, electrochemistry, and energy-related applications.

## Graphical abstract

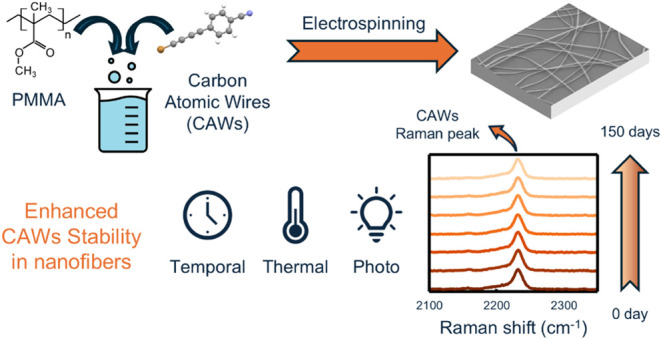

## 1 Introduction

Among the different nanostructures that carbon can assume, carbyne is one of the least investigated. Theoretical models describe carbyne as an infinitely long, one-dimensional wire composed only of sp-hybridized carbon atoms.^1,2^ However, in nature, carbyne-like nanostructures can only exist in finite length, with chain terminations made of different functional groups (–H, −CN, −CH_3_, -phenyl, metals, etc.^3–9^ ); these structures are referred to as carbon atomic wires (CAWs).^1,2^ Depending on the bonding arrangement within the sp-chains, CAWs can be classified into two main categories: cumulenes and polyynes. Cumulenes exhibit consecutive double bonds and are metals in the infinite chain length limit, while long polyynes display semiconducting properties due to their structure, made by alternating single and triple bonds.^1,2,10^

The interest in CAWs arises from their exceptional predicted properties (mechanical, thermal, optical, and electrical) among the best found.^11–14^ Moreover, these properties can be tailored with remarkable precision by manipulating their chemical structure, such as chain length and functional group terminations.^1,2,15^ This high tunability makes carbynes a promising material for developing next-generation materials with tailored properties. However, the stability of CAWs remains a critical challenge, as they tend to undergo cross-linking reactions within each other under exposure to high temperatures (stability drops at around 100 °C), ozonolysis, or light irradiation. These reactions convert sp-hybridized carbon chains to more energetically stable sp^2^-hybridized structures.^16–19^

One of the main strategies to avoid these phenomena is the synthesis of chains with bulky end-groups.^3,14,20–29^ These bulky terminations introduce steric hindrance, reducing the probability of interactions between neighboring chains. Chemical synthesis proves to be a highly effective method for producing CAWs with larger terminations.^14,23,27,29–31^ Another approach to enhance stability is the immobilization of the chains within other nanostructures, such as carbon nanotubes—forming confined carbynes^32–36^ —or within solid matrices, such as nanoparticle assemblies, gels, and polymers.^37–44^ This approach reduces the mobility of CAWs, contributing to the overall material stability. Studies have demonstrated the successful embedding of sp-carbon chains within polymeric matrices through drop-casting or spin-coating techniques.^37,39,44^ However, these methods have exhibited limitations in terms of homogeneity of composition, especially in the center and in the edges of the films.^44^

A new approach to the production of carbyne-based polymeric nanocomposites is electrospinning, a technique that uses electrostatic forces to generate nanoscale fibers from a polymeric solution.^45–48^ Electrospinning provides several advantages over conventional film deposition techniques. First, being the diameters in the nanometric range, materials feature a very high surface-to-volume ratio^46,49,50^ making electrospun membranes perfect for applications that require a large surface area (e.g., hydrogen storage, batteries, supercapacitors).^51–57^ Moreover, electrospinning can be used to create complex composite structures or to align fibers along a direction. This latter capability enables the fabrication of anisotropic materials with different physical properties along the parallel and perpendicular directions, making them valuable for optical devices and other applications.^58–63^ Despite its simple setup, electrospinning is a technique that requires careful control of several parameters to achieve precise control over the morphology and dimensions (i.e., average diameter) of the resulting nanofibers. These parameters can be broadly categorized into ambient parameters (e.g., temperature and humidity), solution parameters (e.g., viscosity, polymer concentration, conductivity, etc.), and process parameters (e.g., applied voltage, flow rate, and needle-to-collector distance). Since these parameters are highly interdependent, it is not always possible to establish a clear correlation between a change in a specific parameter and a variation in fiber morphology or average diameter. A detailed explanation of the role of each electrospinning parameter can be found in several reviews in the literature.^45–48^ In our work, to obtain defect-free fibers with the smallest possible diameter, various applied voltages, flow rates, and needle-to-collector distances were tested to identify the optimal combination of these parameters.

An example of sp-carbon systems within poly­(vinylidene fluoride) (PVDF) nanofibers is reported by Mariappan et al.^64^ By means of low-temperature carbonization via chemical dehydrohalogenation of PVDF, they obtained structures formed by sp and sp^2^ carbon atoms and proved the presence of polyynes with Raman spectroscopy. Our approach is different because we aim to stabilize sp-carbon chains within nanofibers by incorporating a specific class of chemically synthesized halogenated CAWs in the form of polyynes.^4,65,66^ Only a few works investigate the study of CAWs terminated with at least one halogen termination.^67–69^ These systems are of great interest since it has been proven how the choice of the halogen atom and of the chain length is particularly effective in the modification of their optoelectronic and conjugation properties.^69^ Therefore, blending halogenated CAWs with an appropriate polymeric matrix represents a crucial step in the production of nanocomposite materials with tailored properties.

In particular, we embedded three different halogenated CAWs inside poly­(methyl methacrylate) (PMMA) electrospun nanofibers. Each CAW features a benzonitrile group on one end and a halogen atom (Cl, Br, or I) on the other, with a 4-atom polyynic sp-carbon chain connecting them.^4,65,66^ The morphology and diameters of the fibers as well as the influence of incorporating different halogenated CAWs, were investigated using scanning electron microscopy (SEM). The stability of CAWs during electrospinning, the homogeneity of their distribution, and their stability over time, upon heating and photon irradiation were assessed through Raman spectroscopy. CAWs exhibit a distinctive and intense Raman peak (Effective Conjugation Coordinate mode, ECC) in the 1800–2300 cm^–1^ region, absent in all other carbon allotropes.^15,20,32,70–72^ This peak is a reliable marker for identifying sp-hybridized carbon chains. Furthermore, the frequency of the ECC is sensitive to both chain termination and length, enabling the characterization of the chemical structure of the CAWs embedded within the polymeric matrix.^15,20,70^ Our findings revealed that PMMA nanofibers exhibit a more homogeneous CAWs distribution than thin films. Moreover, CAWs embedded within nanofibers effectively preserve their sp-nature over several months, showing a degradation trend comparable to previous studies on the stabilizing effect provided by polymeric films,^37,39,44^ thus demonstrating that the larger surface area of nanofibers is not detrimental to the stability. Additionally, we demonstrated that the nanofiber matrix is also highly effective in enhancing the stability of CAWs under both elevated temperatures and light irradiation. The role of different halogen terminations in affecting the stability has also been thoroughly investigated.

## 2 Methods

Poly­(methyl methacrylate) (PMMA) with *M*_w_ = 996k was purchased from Sigma-Aldrich; *N*,*N*-dimethylformamide (DMF), HPLC grade ≥99.7%, was purchased from Alfa Aesar; halogenated CAWs (in the form of powders) were chemically synthesized following the procedure reported in previous works.^4,66^ These CAWs present a polyynic sp-carbon chain made of 4 carbon atoms that is terminated with a benzonitrile group, on one side, and a halogen atom on the opposite side (see Figure 1). In this work, 3 different halogen terminations have been considered (Cl, Br, and I). We refer to these systems as C_4_X, where X = Cl, Br, or I.

**Figure 1:**
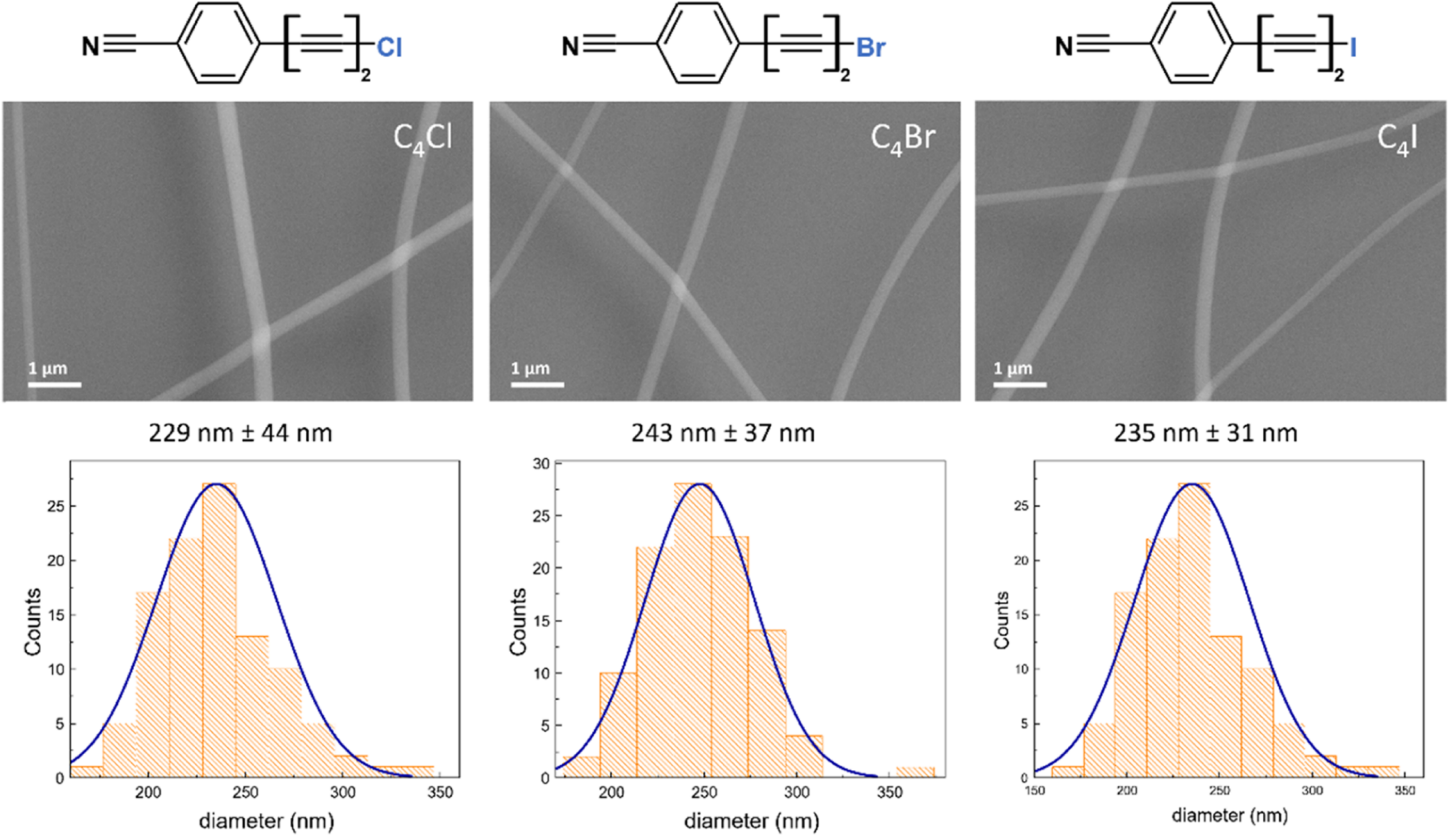
Chemical structure of the C_4_X halogenated CAWs (X = Cl, Br, I) with the relative SEM images and diameter distributions (calculated from approximately 100 diameters) of the electrospun PMMA nanofibers embedding the halogenated CAWs.

Electrospinning was performed using a PMMA solution in DMF (6 wt %) in which C_4_X powder (2 × 10^–2^ M) was dissolved. After solvent evaporation, the C_4_X content in PMMA was 5.8, 7.1, and 8.5 wt % for X = Cl, Br, and I, respectively. Briefly, PMMA was weighted and dissolved inside DMF stirring the solution at 60 °C until the polymer was completely dissolved (i.e., approximately 4–5 h). When the polymeric solution was cooled down to room temperature, the weighted amount of C_4_X powder was put inside the solution and stirred for 3 h until a homogeneous solution was obtained. The electrospun membranes were obtained by loading the feed solution in a 2.5 mL syringe with a 22-gauge needle (Hamilton Gastight model 1002 TLL), which was then placed on an infusion pump (KDS Scientific, model series 200). The electrospinning process was carried out using a horizontal setup. Voltage was applied to the needle by a high-voltage power supply (Spellman SL30P300). The optimal parameters to obtain fibers with no defects and a small average diameter were obtained by testing 3 different applied voltages (9, 12, and 15 kV), 3 different pump-rates (0.05, 0.1, and 0.2 mL/h), and 3 different needle-to-collector distances (15, 20, and 25 cm). Images of the fibers were obtained with scanning electron microscopy (SEM) using a Zeiss Supra 40 Field Emission Scanning Electron Microscope; the mean diameter and the standard deviation of the fibers were measured using ImageJ software (the number of diameters considered for each sample was approximately 80–100). Analyzing the SEM images, we observed how the fibers with the best homogeneity and a smaller diameter were obtained with 12 kV, 0.2 mL/h, and 20 cm. These parameters were set as the standard to obtain electrospun samples for Raman measurements. Films of the same polymeric solutions were obtained by drop-casting these solutions onto silicon substrates and gently evaporating the solvent. Raman spectra were obtained with a Renishaw inVia Raman microscope with a diode-pumped solid-state laser (λ = 532 or 660 nm). All the spectra were acquired with 532 nm laser excitation using 3.5 mW power and doing 5 accumulations of 10 s each; spectra were acquired in different points of each sample and the signals were averaged to compensate for possible local inhomogeneity of the samples. The photodegradation spectra were acquired both with the 532 nm (35 mW) and the 660 nm laser (37.7 mW) doing one accumulation of 10 s; spectra were obtained all in the same point to observe the evolution of the sample under light irradiation.

## 3 Results and Discussion

To investigate the impact of different electrospinning parameters on fiber morphology and average diameter, we electrospun solutions of pure PMMA in DMF (6 wt %) at distinct needle-to-collector distances (*D* = 15, 20, and 25 cm), applied voltages (*V* = 9, 12, and 15 kV), and flow rates (ϕ = 0.05 0.1, and 0.2 mL/h). SEM images of all samples are presented in Figures S1–S3. These images reveal an excellent fiber morphology without defects for all parameter combinations. In Figure S4, the average diameters of all the electrospun samples are reported. All fibers exhibit an average diameter, ⟨*d*⟩, in the 200–400 nm range, with applied voltage and needle-to-collector distance playing a major role in affecting this parameter, while the flow rate has only a minor effect. However, the diameter distributions of all tested nanofibers are quite similar, making it difficult to define a clear trend as a function of the different electrospinning parameters.

In the following, all fibers were electrospun using the optimized parameters of *D* = 20 cm, *V* = 12 kV, and ϕ = 0.2 mL/h, which yield fibers with the smallest mean diameter and standard deviation (⟨*d*⟩ = 220 ± 28 nm, as shown in Figure S4). Figure 1 presents SEM images of fibers containing the three halogenated sp-carbon chains, along with their corresponding diameters distributions. It is evident that the morphology and diameter of the fibers are not significantly affected by the presence of CAWs, with only a slight increase of 10–20 nm compared to pure PMMA fibers. This can be attributed to the high polarity and high boiling point of the solvent used (DMF), which are hardly affected by the presence of CAWs.

After electrospinning solutions containing PMMA and halogenated CAWs for 30 min, Raman spectroscopy was employed to characterize the so-obtained membranes. Figure 2a compares the Raman spectra of the as-prepared samples with those of pure PMMA fibers and pure powders of halogenated CAWs. The bottom panel reveals that the polymer is characterized mainly by three strong peaks in the 2800–3100 cm^–1^ region associated with C–H stretching vibrations^73^ and a minor peak at 1730 cm^–1^ attributable to C=O stretching.^74^ The central panel presents the spectra of the three C_4_X CAWs, which exhibit two dominant peaks: the phenyl stretching mode at approximately 1600 cm^–1^ and the ECC mode in the 2200–2250 cm^–1^ region.^69^ A weak peak related to the CN group of the C_4_X systems is also present at 2233 cm^–1^. It is only visible in C_4_I since for C_4_Cl and C_4_Br, it overlaps with the respective ECC peaks. A detailed investigation of the Raman spectra of C_4_X, including the assignment of each mode using DFT calculations, is presented in our previous work.^69^ The top panel indicates that the characteristic peaks of PMMA and of the halogenated CAWs are still detectable, even in CAWs/PMMA nanofibers. This suggests that, despite the electrospinning process’s exposure to high voltages and strong stretching forces, the highly reactive CAWs are not degraded and are successfully embedded within the nanofibers. Further evidence that halogenated CAWs are effectively embedded within the nanofibers is provided by EDX analysis performed on the three nanofiber samples containing CAWs (Figure S5), which confirms the presence of Cl, Br, and I atoms in the electrospun membranes with an atomic percentage of approximately 1%. Figure 2b highlights the ECC region. As previously documented in the literature,^69^ moving from Cl to I results in an enhanced electron-donating capability of the halogen termination, leading to an increase in sp-carbon chain π-electrons conjugation. This is reflected as a decrease in the bond length alternation (BLA) (i.e., the difference in bond lengths for adjacent bonds^1^), which in turn reflects a reduction in the optical energy gap and a shift toward lower wavenumbers of the ECC peak position. Hence, a correlation exists between the vibrational characteristics (i.e., the Raman ECC frequency) and the chemical structure of the CAWs.^75^ Figure 2b demonstrates the variation in the ECC frequency as the halogen termination is changed. This shift is observed in both powder and nanofiber-embedded CAWs, indicating that the halogen atom’s influence on CAWs properties is still present even when these systems are embedded in polymeric matrices. Interestingly, the ECC peak positions for the three CAWs are nearly identical in both powder and fiber forms, suggesting that embedding inside PMMA nanofibers has a negligible influence on the optoelectronic properties of CAWs. This result is of particular importance since in previous works^69^ we observed a shift of the ECC peak when passing from powders to isolated molecules in solution. This shift is due to intermolecular interactions, in particular to halogen bonds, occurring in the CAWs crystals (i.e., powders) but not present when CAWs behave as isolated molecules (i.e., in solutions). These interactions affect the π-electrons system, leading to a lowering of the ECC frequency.^69^ In analogy with the behavior observed in CAWs solutions, if CAWs are embedded within nanofibers as individual isolated molecules, meaning that the intermolecular interactions typical of the crystalline phase were completely lost, the ECC peak would be expected to shift compared to that of powders, due to the absence of these specific intermolecular interactions. Two different phenomena can explain why the expected shift is not observed: (i) differently from the solution, the PMMA matrix provides sites for specific intermolecular interactions like in the crystal, allowing halogen bonds formation with CAWs where the PMMA ester group acts as acceptor;^76–78^ (ii) the CAWs inside the nanofibers are not individual isolated molecules dispersed in the matrix. Instead, due to the strong halogen bond between pairs, small aggregates, with a supra-molecular arrangement similar to that of the crystal phase may still persist—as previously observed in other works^79,80^ —thus retaining the intermolecular halogen bond interactions between CAWs pairs and resulting in no shift of the ECC frequency.

**Figure 2:**
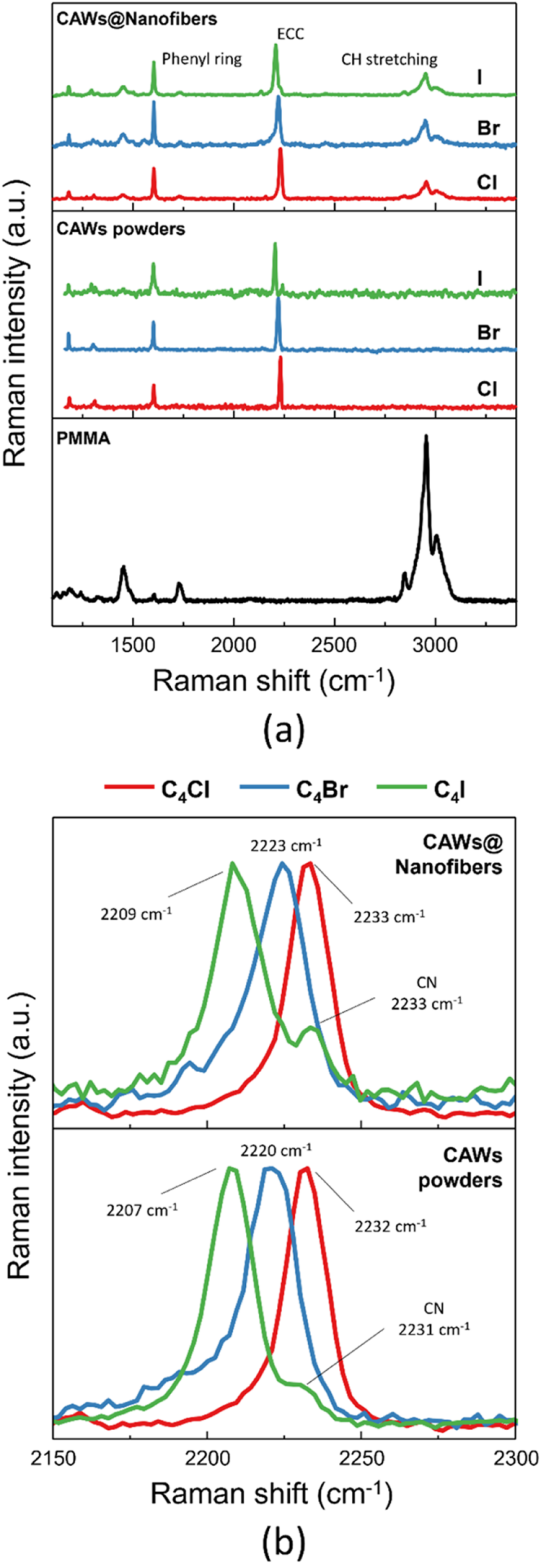
(a) In the top panel, Raman spectra of electrospun nanocomposites embedding C_4_X halogenated CAWs (X = Cl, Br, I). In the central panel, Raman spectra of powders of the same CAWs. In the bottom panel, Raman spectrum of PMMA powder. (b) Enlargement of the ECC region of the top and central panels of (a).

Previous research has demonstrated that polymeric composites containing CAWs, prepared using film deposition techniques such as drop-casting, exhibit a nonhomogeneous ratio of the Raman signal intensity between the CAWs and the polymer matrix.^44^ This inconsistency is due to the “coffee-ring effect”, which leads to uneven deposition of the composite material on the edges and center of the samples. To evaluate the uniformity of CAWs/PMMA composites, Raman spectroscopy was employed to characterize both the electrospun membranes and the drop-cast films obtained from the same solutions. Raman spectra were obtained from multiple points across the samples moving from the center to the edges and collecting spectra each of 2 mm. Homogeneity was assessed considering the ratio (*R* = *A*_ECC_/*A*_polymer_) of the integral of the CAWs ECC band and the integral of the structured CH-stretching band of PMMA at different points of the electrospun membranes and of drop-cast films. The comparisons of fibers and films are presented in Figures 3 and S6. In films, the ratio decreases significantly from the center to the edges, indicating a poor homogeneous distribution of CAWs across the sample. In contrast, fibers exhibit an almost constant ratio throughout the sample, with values near the edges comparable to those at the center, suggesting a much more uniform CAWs distribution. These results can be attributed to the different evaporation dynamics of the two techniques. In drop casting, solvent evaporation occurs gradually and unevenly, starting from the edges and progressing toward the center. In regions with slower evaporation rates (i.e., the center), CAWs tend to accumulate, leading to higher local concentrations and variations in the CAWs/PMMA ratio across the film. Conversely, in electrospinning, solvent evaporation is extremely fast, and fibers are fully dried upon collection when an appropriate needle-to-collector distance is maintained. Additionally, the random deposition of fibers ensures that both the edges and the center of the substrate are covered simultaneously. As a result, even if CAWs might exhibit a gradient within individual fibers, their overall distribution across the electrospun membrane remains highly homogeneous. These observations are further confirmed by considering the percentage variation in the CAWs/PMMA ratio across the different points. Indeed, for fibers, the variations are 4.8, 5.5, and 3.6% for C_4_Cl, C_4_Br, and C_4_I, respectively; while, in the films, the variation is 10.2, 13.9, and 17.3% for C_4_Cl, C_4_Br, and C_4_I, respectively. In the case of films, these values are more than doubled, demonstrating how the homogeneity of the signal, and consequently the homogeneity of CAWs distribution within the samples, is significantly enhanced with the electrospinning process compared to drop casting.

**Figure 3:**
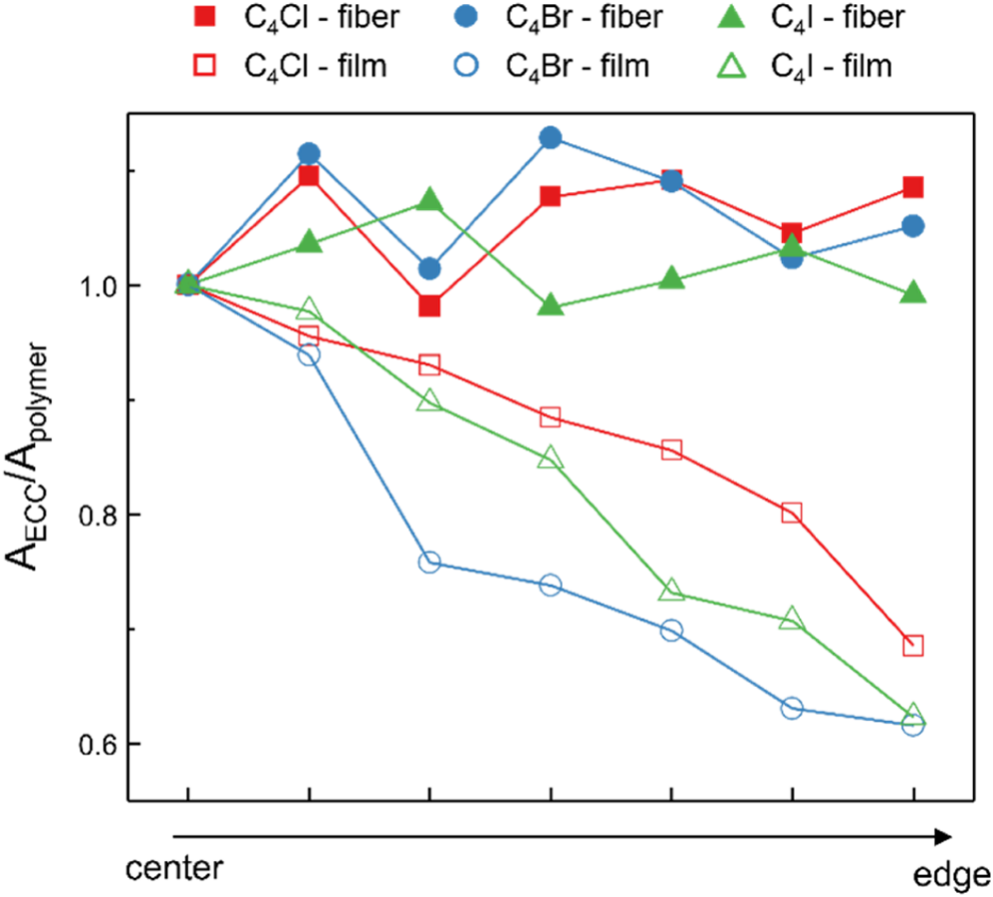
Ratio between the areas of the CAWs ECC band and the PMMA CH-stretching band for electrospun membranes (solid symbols) and drop-cast films (hollow symbols), measured at different positions across the samples, from the center to the edges.

Previous works have demonstrated the stability of CAWs embedded in polymeric films over time.^37,39,44^ These studies observed degradation in the first days of storage, followed by almost constant Raman signal preservation for several months. This behavior can be attributed to detrimental interactions between oxygen or other environmental agents and the CAWs found on the surface layers of the films. Surface layers are more exposed to degradation processes and, therefore, undergo significant degradation in the initial days, leading to a rapid Raman signal reduction. Subsequently, the CAWs within the inner regions of the films are more shielded and less likely to interact with environmental molecules, resulting in improved stability and a much slower signal reduction. In this work, we investigated the stability over time of halogenated CAWs embedded inside of nanofibers. Due to the high surface-to-volume ratio achieved through electrospinning, these nanofibers experience increased exposure to the environment. We aim to understand whether this feature can significantly affect the stability of CAWs compared to CAWs powders and CAWs/PMMA films. To address this question, we monitored the Raman signal of PMMA + halogenated CAWs electrospun membranes over several months, observing changes in the spectra, specifically the characteristic ECC peak. The results for the three polymeric composites are shown in Figures 4a and S7 and are compared to the stability of the powders stored under identical conditions (Figure S8). In the case of powders, rapid degradation occurs and the ECC signal disappears within 4 days (X = Cl and Br) or 11 days (X = I). The higher stability of C_4_I can be attributed to the lower electronegativity of this halogen termination with a consequent reduction of the C-X bond polarization. Indeed, the higher polarity of the C-X bond (X = Cl, Br) makes easier the occurrence of reactions like cross-linking, nucleophilic attacks, and cycloadditions from external agents, thus lowering the stability of C_4_Cl and C_4_Br. Differently, electrospun membranes exhibited significantly enhanced stability, with the ECC signal still readily detectable for all three nanocomposites, even after six months of storage. This remarkable stability is a clear confirmation of the protective capabilities of a polymeric matrix, effectively shielding the sp-carbon chains from detrimental environmental factors. The shielding action not only minimizes the mobility of the CAWs, thereby reducing the likelihood of interchain interactions and cross-linking, but also effectively isolates them from the surrounding environment, minimizing their exposure to harmful agents such as oxygen. The stabilizing effect of the electrospun nanofibers is further investigated by considering the evolution over time of the ratio (*R* = *A*_ECC_/*A*_polymer_) between the integral of the CAWs ECC band and that of PMMA CH-stretching band (Figure 4b). C_4_Br and C_4_I show an almost constant value of *R* throughout the investigated period, while C_4_Cl exhibits lower stability (due to the higher electronegativity of the chlorine atom, as previously discussed for powders) with rapid degradation in the first days, followed by gradual stabilization of the degradation process. However, we confirm that all three nanocomposites demonstrate good stability, maintaining a good ECC Raman signal even after several months. As a comparison, Figure 4c also reports the ratio between the CAWs phenyl band and the PMMA CH-stretching bands. This ratio remains very stable over all of the tested times, indicating that the benzonitrile termination is not affected by degradation. Instead, degradation mainly occurs along the sp-C chain, leading to a variation in the ECC peak intensity over time (Figure 4b).

**Figure 4:**
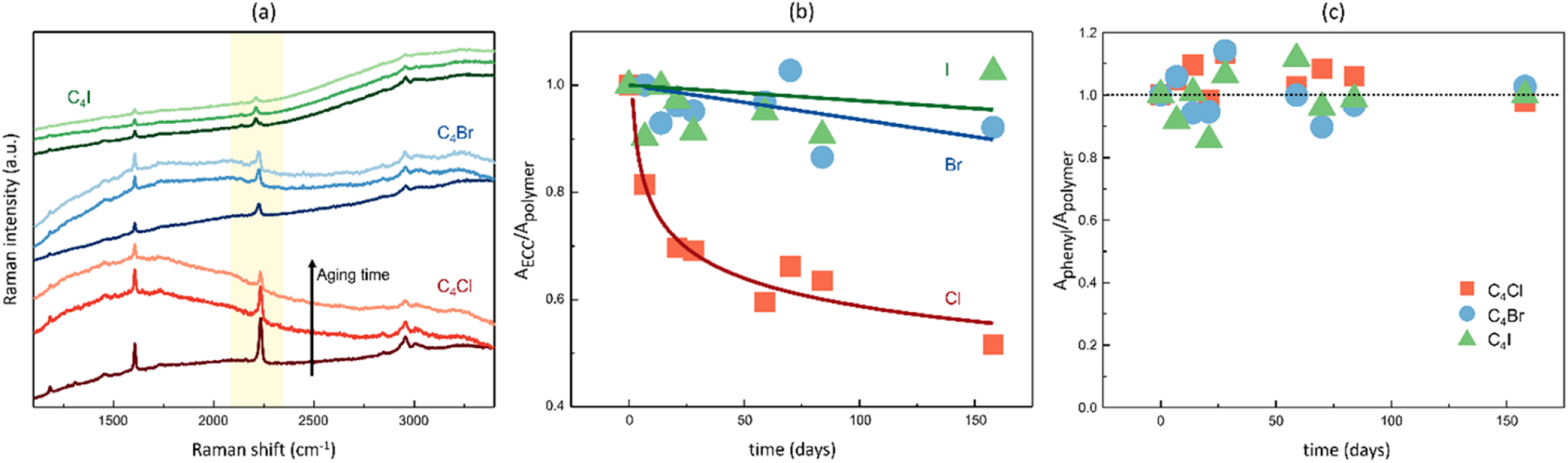
(a) Raman spectra of the electrospun membranes containing C_4_X halogenated CAWs (X = Cl, Br, I) measured at 0, 7, and 158 days from deposition (from dark to light, respectively) after electrospinning. The ECC region is highlighted in yellow. (b) Time evolution of the ratio between the areas of the CAWs ECC band and the PMMA CH-stretching band for the electrospun membranes containing C_4_X halogenated CAWs (X = Cl, Br, I). (c) Time evolution of the ratio between the areas of the CAWs phenyl band and the PMMA CH-stretching band for the same membranes.

Other differences between the three membranes arise considering the background of the Raman spectra at different times (Figures 4a and S7). The Raman signal for iodine-terminated CAWs remained almost constant throughout the storage period, while for chlorine and bromine, a broad background developed in the 1600 cm^–1^ region. The higher stability of iodine-terminated CAWs impedes the formation of sp^2^-hybridized byproducts, the primary contributors to the observed broad background, and results in a more stable ECC Raman signal. The formation of the byproducts occurs mainly in the first days (i.e., associated with degradation of CAWs in the superficial layers more exposed to the environment), after which further degradation is minimal. Interestingly, this degradation behavior is consistent with what has been observed in previous works on thin films,^37,39,44^ and has been compared with the degradation occurring in thin films obtained through drop-casting the same PMMA-halogenated carbon chain solutions (Figure S9). Thin films embedding C_4_I present a very constant Raman signal over time, while films embedding C_4_Cl and C_4_Br experienced the same background degradation observed for nanofibers in the first week, followed by an almost constant ECC Raman signal for longer periods. These results indicate that nanofibers offer the same level of stability as film matrices despite their significantly higher surface-to-volume ratio. Therefore, electrospinning emerges as the most suitable technique for fabricating stable CAWs-based materials, particularly in applications where a high surface-to-volume ratio is crucial (e.g., supercapacitors,^55–57^ next-generation batteries,^51,52^ hydrogen storage,^53,54^ etc.).

Figures 5a and S10 represent Raman spectra of nanofibers exposed to thermal treatment (90 °C) for different times up to 48 h (2880 min). The observed thermal stability trend mirrors the stability pattern over time, at ambient temperature, exhibiting enhanced stability with decreasing halogen electronegativity, from Cl to I. In particular, iodine-terminated fibers retain the characteristic ECC peak even after 2 days of heating, while chlorine and bromine derivatives exhibit almost complete signal attenuation after 20 and 48 h, respectively. Additionally, for Cl and Br samples, a broader background emerges with increasing heating time, which is attributed to the formation of byproducts containing sp^2^-hybridized carbon atoms. This background rise coincides with a decrease in signal-to-noise ratio. These findings align with the observed stability over time, suggesting that cross-linking between adjacent sp-carbon chains and interactions with external agents play a crucial role also in thermal degradation. Moreover, the thermal stability analysis reveals a faster degradation rate compared to stability over time. This acceleration can be attributed to the enhanced kinetic processes facilitated by higher temperatures and the proximity to the glass transition temperature (*T*_g_) of the PMMA matrix (in the range of 95–125 °C^81^). In this condition, the polymeric chains shielding the halogenated CAWs exhibit increased free volume and chain mobility, promoting two detrimental phenomena: easier penetration of oxygen and other molecules into the fibers and a reduced locking of the CAWs, allowing for their enhanced mobility within the matrix. These factors directly contribute to the accelerated degradation process by increasing the extent of oxidation and cross-linking, respectively. Interestingly, Raman spectra of C_4_Br and C_4_I nanofibers exhibit the formation of a secondary peak (indicated with ° in Figure 5a) in the ECC region with lower frequencies than the ECC one (2189 and 2131 cm^–1^ for Br and I, respectively). The intensity of the secondary peak increases with heating time (bottom panel of Figure 5b), suggesting that it is generated from the formation of new molecular species containing sequences of sp-carbon atoms (as indicated by their peak in the CAWs ECC region), whose generation is promoted by temperature. Interestingly, in our previous work,^69^ we measured ECC frequencies at 2187 and 2132 cm^–1^ for similar halogenated CAWs with the same termination groups (i.e., benzonitrile and halogen atoms) but with longer sp-C chains—specifically, 6 and 8 sp-C atoms for Br and I, respectively. The Raman ECC frequencies of these systems, termed C_6_Br and C_8_I, are very close to the secondary peaks arising during the thermal process, suggesting that a change of the chemical structure favored by temperature might occur, leading to CAWs with increased chain lengths. As a hypothesis, thermal energy could induce the cleavage of some CAWs close to the end groups, leading to the formation of radical fragments. Fragments originating from different parent CAWs may subsequently combine to form new CAWs with greater sp-C chain lengths (i.e., higher conjugation and a red shift of the ECC peak position). Examples of the possible structures are shown in Figure S11. The evolution in time of CAWs ECC bands (normalized with respect to the PMMA CH-stretching bands) has been reported in Figure 5b (top panel). The increase in stability from chlorine to iodine is observable. Membranes embedding C_4_Cl and C_4_Br show a decrease in the intensity of the ECC peak with increasing heating time, while the ECC peak of C_4_I appears to be almost constant.

**Figure 5:**
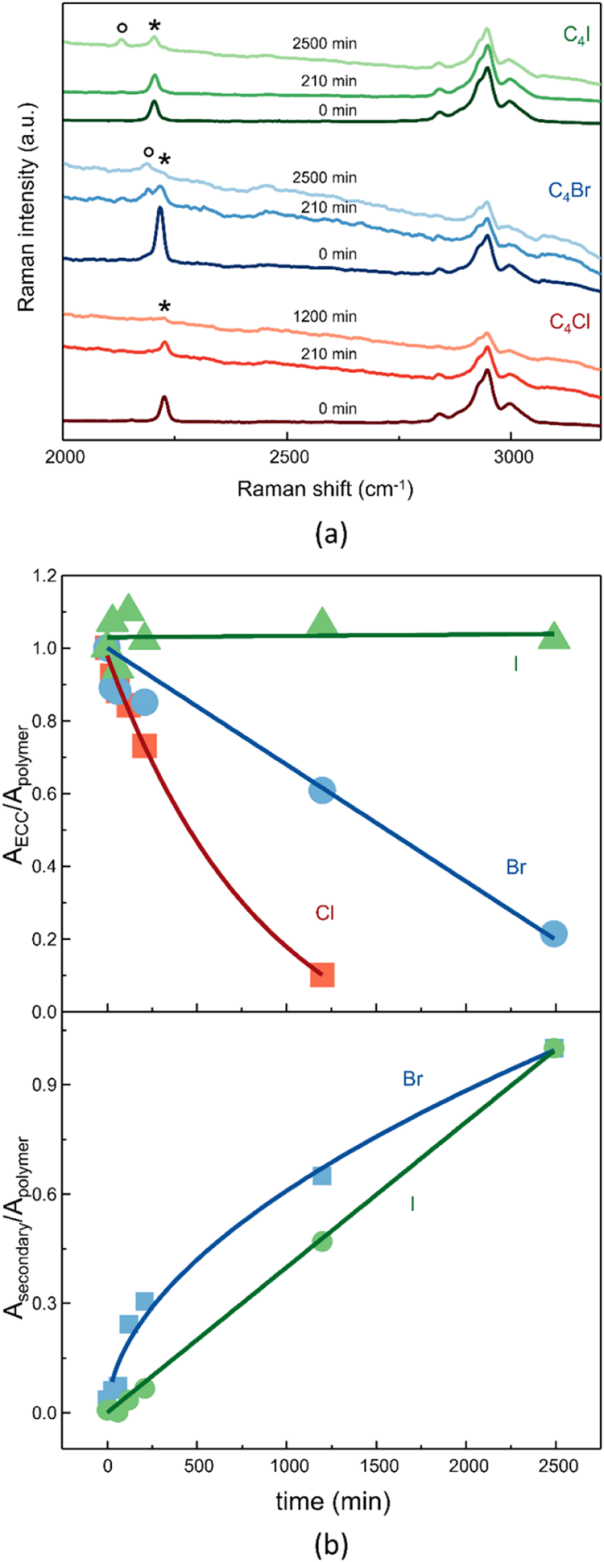
(a) Raman spectra of the electrospun membranes containing C_4_X halogenated CAWs (X = Cl, Br, I) measured after heating at 90 °C for different times. The symbols * and ° indicate the position of the CAWs ECC and the secondary peak, respectively. In the top panel of (b), the time evolution of the ratio between the areas of the CAWs ECC band and the PMMA CH-stretching band for the electrospun membranes containing C_4_X halogenated CAWs. In the bottom panel of (b), the evolution over time of the ratio between the areas of the secondary band and the PMMA CH-stretching band for the electrospun membranes containing C_4_X halogenated CAWs.

In addition to the effect of the decreasing polarity of CX bonds with the decreasing electronegativity of the halogen atom, the significant stability of C_4_I nanofibers can be attributed to the increasing strength of halogen bonds (from Cl to I^82^) which the CAWs halogen terminations can form. Iodine-terminated CAWs can form stronger halogen bonds with the ester groups of the PMMA matrix, resulting in better immobilization and stabilization of C_4_I during the thermal process with respect to C_4_Cl and C_4_Br. Moreover, for both C_4_Br and C_4_I, the area of the secondary peak is increasing with longer times, indicating that even for C_4_I nanofibers, the degradation process is occurring. Even though the stability of the halogenated CAWs embedded inside nanofibers is much lower at higher temperatures than at room temperature, comparing the stability of C_4_X powders and the electrospun membranes exposed to the same high temperatures, we observe an increase in stability promoted by the presence of the polymer. The powders’ ECC peaks are no longer detectable after 30, 90, and 120 min for C_4_Cl, C_4_Br, and C_4_I, respectively (Figure S12). These times are shorter than those previously reported for nanofibers (20 and 48 h for C_4_Cl and C_4_Br, respectively. C_4_I presents the ECC signal even after 48 h), indicating that the shielding from environmental agents and the reduction in CAWs mobility provided by a solid polymeric matrix can also be effective in increasing the thermal stability of these systems. This is further supported by SEM images of the nanofibers after exposure to different temperatures (i.e., 50, 70, 90, and 130 °C) for 4 h (Figure S13), following an approach previously reported in the literature.^83^ The images show that at temperatures below the glass transition temperature of PMMA (ranging from 95 to 125 °C^81^), the nanofibers retain their morphological structure well with no significant variations in the average diameter. However, at temperatures above the glass transition (i.e.,130 °C), the nanofibers appear to swell and lose their original morphology. The good CAWs stabilization provided by polymeric nanofibers at 90 °C can therefore be attributed to the fact that at this temperature, nanofibers preserve their structure, thus continuing to act as an effective barrier against environmental agents (e.g., oxygen).

To gain a comprehensive understanding of the stability of these nanocomposites, we also investigated their photodegradation resistance. Figures 6a and S14 report the stability of nanofibers containing the three halogenated CAWs upon exposure to 532 nm laser radiation (35 mW of power) for different times. The same irradiation protocol was employed for the powders of these CAWs (Figures 6b and S15). In the case of powders, rapid degradation was observed for all three CAWs: C_4_Br and C_4_I completely decomposed after 1 min of light exposure, while C_4_Cl exhibited a 25% of its initial ECC intensity after 2 min of irradiation. On the contrary, embedding the same CAWs within electrospun polymeric matrices significantly enhanced their photostability. For C_4_Br and C_4_I, the ECC signal was reduced but remained detectable after 2 min of light irradiation (43% and 20% of their respective initial values), while C_4_Cl retained an ECC band intensity of 80% after 2 min of irradiation. These findings highlight the protective effect of polymeric matrices against photodegradation. The improved photostability can be attributed to the isolation and immobilization of CAWs within the polymeric host.

**Figure 6:**
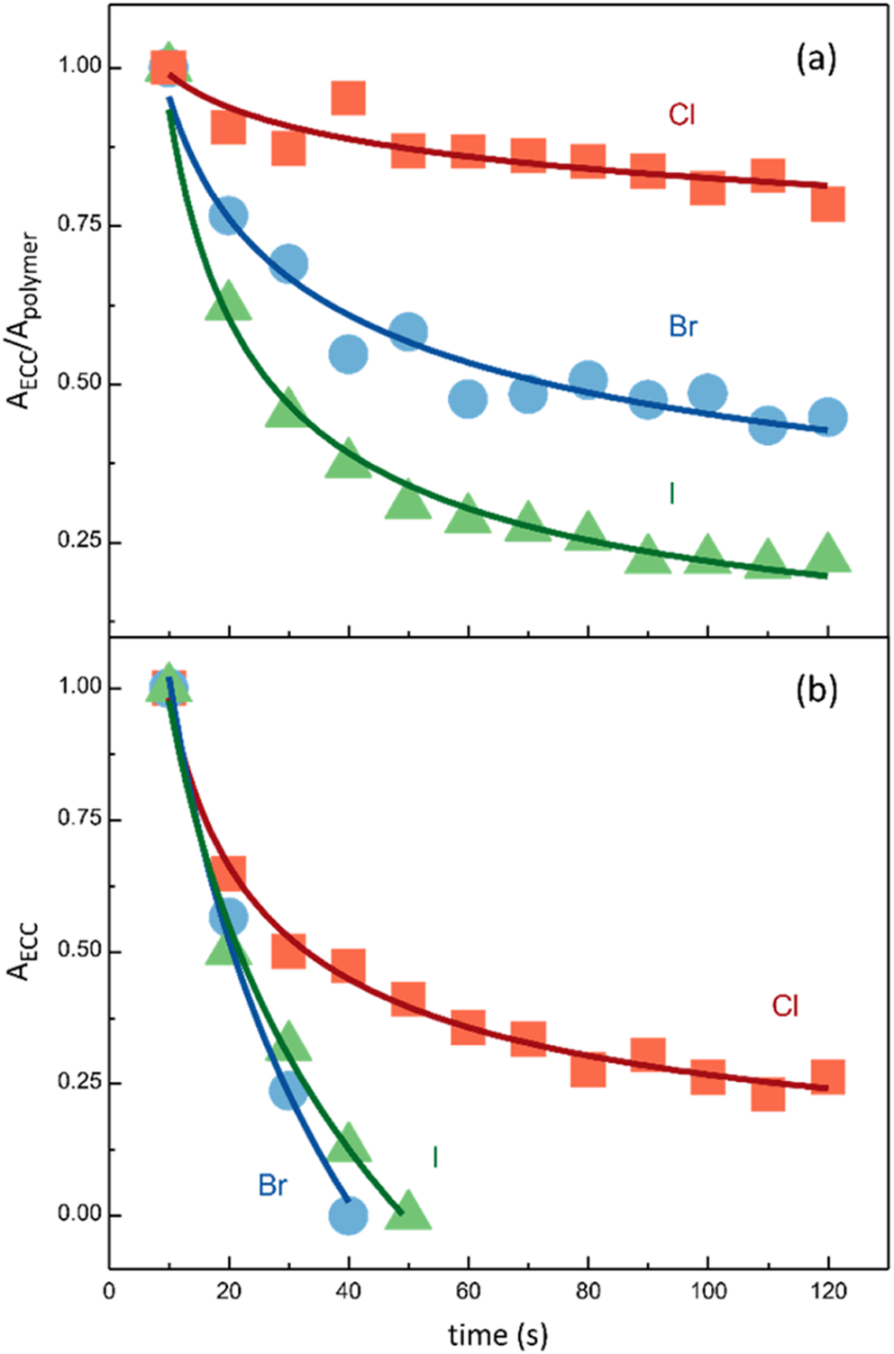
Evolution of the CAWs ECC Raman bands areas for the electrospun membranes (a) and the powders (b) of the C_4_X halogenated CAWs (X = Cl, Br, I) after exposure to light irradiation (532 nm) for different times. In (a), the ratio between areas of the CAWs ECC band and the PMMA CH-stretching band is considered. In (b), the area of the CAWs ECC band is considered.

As discussed earlier for time and thermal stability, high electronegativity halogen terminations (i.e., Cl) are less effective in preventing time and thermal degradation. Therefore, halogen electronegativity cannot explain why C_4_Cl exhibits greater photostability compared to C_4_Br and C_4_I. This contrary trend suggests that the primary physical mechanism governing photodegradation differs from those affecting stability over time and under heating. To better comprehend the role of halogen termination on the photodegradation process, we performed a similar laser exposure experiment using a 660 nm wavelength (37.7 mW) on the same electrospun nanocomposites (Figures S16 and S17). Interestingly, despite the laser power being comparable to that of previous experiments with irradiation at 532 nm, in this case, we observe a very constant ECC signal for all the three samples, indicating minimal photodegradation. Nonlinear optical phenomena can explain such behavior. For instance, a two-photon transition with absorption of photons at 532 nm corresponds to an energy supply of Δ*E* = 4.66 eV (λ = 266 nm), in the spectral region where the vibronic sequence associated with the HOMO–LUMO transition of C_4_X species is observed (Figure S18). A photoexcited state, in turn, could promote the homolytic cleavage of the C–X bonds, leading to the formation of radicals.^84–86^ Photodegradation stemming from two-photon absorption is a known phenomenon and was reported for some dyes used in two-photon microscopy.^87^ The photostability trend (Cl > Br > I) correlates with the bond dissociation energies of C–Cl (338 kJ/mol), C–Br (280 kJ/mol), and C–I (232 kJ/mol). Since more energy is required to break the C–Cl bond, it is more stable than the C–Br and C–I bonds. Therefore, the homolytic cleavage mechanism can explain why C_4_Cl is the most stable CAW analyzed here. Lower energy photons (660 nm) are unsuitable for promoting a two-photon transition (Δ*E* = 3.76 eV, namely, 330 nm, below the CAWs absorption edge), and thus they cannot induce a photoassisted C–X cleavage for all the C_4_X systems, explaining why the photodegradation with the 660 nm laser was almost zero for all the analyzed samples. Another effect that can contribute to the greater stability of C_4_Cl is the formation of photoinduced radicals favored by the presence of halogen bonds. As reported in the literature,^88^ the formation of a halogen bond can weaken the C–X bond, making it easier to break when photoexcited. Since iodine can form halogen bonds, either with the PMMA matrix or with other C_4_I molecules,^69^ which are stronger compared to the halogen bonds of chlorine and bromine, the corresponding C–X bond will be the most weakened, making C_4_I the system most susceptible to photodegradation.

## 4 Conclusions

In conclusion, our study focused on understanding the influence of various electrospinning parameters on the morphology and diameter of poly­(methyl methacrylate) (PMMA) fibers, revealing nontrivial relationships among needle-to-collector distance, applied voltage, and pump rate. Despite the complexity of the electrospinning process, we successfully identified optimal conditions (*D* = 20 cm, *V* = 12 kV, ϕ = 0.2 mL/h) resulting in PMMA fibers with the smallest average diameters and standard deviations (*d* = 220 ± 28 nm). Incorporation of halogenated CAWs into the electrospun PMMA fibers had minimal effect on fiber morphology, with only a slight diameter increase, attributed to a good compatibility between the PMMA matrix and the halogenated CAWs.

Raman spectroscopy confirmed the successful embedding of CAWs within the nanofibers, highlighting the stability of CAWs even under the electrospinning process. Compared with traditional film deposition, electrospinning ensured a more homogeneous CAWs distribution, overcoming the “coffee-ring effect” seen in drop-cast films.

Moreover, halogenated CAWs electrospun within nanofibers revealed remarkable resistance to degradation over time, demonstrating the potential of these materials for long-term applications. Additionally, exposure to high temperatures highlighted the influence of halogen termination on degradation kinetics, giving insight into the relationship between CAWs termination groups and thermal stability, with low electronegativity terminations (i.e., I) providing higher stability due to the reduced polarization of the C–X bond, which is reflected in an increased chemical stability. Furthermore, the presence of a polymeric matrix significantly enhanced the photostability of CAWs, offering a protective environment against degradation induced by laser irradiation. The role of halogen termination in this context was opposite to that in the case of time and thermal stability, indicating that the physical mechanisms governing photodegradation and chemical modifications over time or at high temperatures are different. Photodegradation behavior was explained based on the photoinduced homolytic cleavage of the C–X bond.

Our work explored the promising applications of electrospun nanofibers incorporating halogenated CAWs, highlighting their advantages over traditional thin films. The high homogeneity achieved through electrospinning addresses the issues of nonuniformity common in thin-film deposition techniques, ensuring an even distribution of halogenated CAWs within the nanofibrous membranes. Additionally, electrospun nanofibers offer a significant advantage with their enhanced surface-to-volume ratio. These features, along with the observed stability of CAWs within the nanofibrous structure, make these materials promising candidates for electronic, electrochemical, and other energy-related applications such as transistors, batteries, and supercapacitors, paving the way for future research.

## Supplementary Material



## Notes

SEM/EDX analysis of the electrospun nanofiber diameters, Raman spectra, and UV–vis absorption spectra (PDF)

## References

[1] Casari C. S., Tommasini M., Tykwinski R. R., Milani A. (2016). Carbon-atom wires:
1-D systems with tunable properties. Nanoscale.

[2] Casari C. S., Milani A. (2018). Carbyne: From the elusive
allotrope to stable carbon
atom wires. MRS Commun..

[3] Cataldo F., Ursini O., Milani A., Casari C. S. (2018). One-pot synthesis
and characterization of polyynes end-capped by biphenyl groups (α,ω-biphenylpolyynes). Carbon.

[4] Gulia N., Pigulski B., Szafert S. (2015). Palladium End-Capped
Polyynes via
Oxidative Addition of 1-Haloalkynes to Pd­(PPh_3_)_4_. Organometallics.

[5] Cataldo F. (2004). Polyynes:
A new class of carbon allotropes. About the formation of dicyanopolyynes
from an electric arc between graphite electrodes in liquid nitrogen. Polyhedron.

[6] Hu F., Zeng C., Long R., Miao Y., Wei L., Xu Q., Min W. (2018). Supermultiplexed optical imaging and barcoding with
engineered polyynes. Nat. Methods.

[7] Ramadhan A., Wesolowski M., Wakabayashi T., Shiromaru H., Fujino T., Kodama T., Duley W., Sanderson J. (2017). Synthesis
of hydrogen- and methyl-capped long-chain polyynes by intense ultrashort
laser pulse irradiation of toluene. Carbon.

[8] Kendall J., McDonald R., Ferguson M. J., Tykwinski R. R. (2008). Synthesis
and solid-state structure of perfluorophenyl end-capped polyynes. Org. Lett..

[9] Gao Y., Tykwinski R. R. (2022). Advances in Polyynes to Model Carbyne. Acc. Chem. Res..

[10] Tongay S., Senger R. T., Dag S., Ciraci S. (2004). Ab-initio
Electron
Transport Calculations of Carbon Based String Structures. Phys. Rev. Lett..

[11] Liu M., Artyukhov V. I., Lee H., Xu F., Yakobson B. I. (2013). Carbyne
from first principles: Chain of C atoms, a nanorod or a nanorope. ACS Nano.

[12] Zhu Y., Bai H., Huang Y. (2016). Electronic Property Modulation of One-Dimensional Extended
Graphdiyne Nanowires from a First-Principle Crystal Orbital View. ChemistryOpen.

[13] Wang M., Lin S. (2016). Ballistic Thermal Transport in Carbyne and Cumulene with Micron-Scale
Spectral Acoustic Phonon Mean Free Path. Sci.
Rep..

[14] Tykwinski R. R., Chalifoux W., Eisler S., Lucotti A., Tommasini M., Fazzi D., Del Zoppo M., Zerbi G. (2010). Toward carbyne: Synthesis
and stability of really long polyynes. Pure
Appl. Chem..

[15] Milani A., Tommasini M., Russo V., Bassi A. L., Lucotti A., Cataldo F., Casari C. S. (2015). Raman spectroscopy as a tool to investigate
the structure and electronic properties of carbon-atom wires. Beilstein J. Nanotechnol..

[16] Cataldo F. (2006). Stability
of polyynes in air and their degradation by ozonolysis. Polym. Degrad. Stab..

[17] Heymann D. (2005). Thermolysis
of the polyyne C_8_H_2_ in hexane and methanol:
Experimental and theoretical study. Carbon.

[18] Casari C. S., Bassi A. L., Ravagnan L., Siviero F., Lenardi C., Piseri P., Bongiorno G., Bottani C. E., Milani P. (2004). Chemical and
thermal stability of carbyne-like structures in cluster-assembled
carbon films. Phys. Rev. B.

[19] Cataldo F. (2004). Polyynes production
in a solvent-submerged electric arc between graphite electrodes. III.
Chemical reactivity and stability toward air, ozone, and light. Fullerenes, Nanotubes Carbon Nanostruct..

[20] Milani A., Lucotti A., Russo V., Tommasini M., Cataldo F., Li Bassi A., Casari C. S. (2011). Charge
transfer
and vibrational structure of sp-hybridized carbon atomic wires probed
by surface enhanced raman spectroscopy. J. Phys.
Chem. C.

[21] Rivelino R., dos Santos R. B., de Brito Mota F., Gueorguiev G. K. (2010). Conformational
Effects on Structure, Electron States, and Raman Scattering Properties
of Linear Carbon Chains Terminated by Graphene-Like Pieces. J. Phys. Chem. C.

[22] Milani A., Tommasini M., Barbieri V., Lucotti A., Russo V., Cataldo F., Casari C. S. (2017). Semiconductor-to-Metal Transition
in Carbon-Atom Wires Driven by sp^2^ Conjugated End Groups. J. Phys. Chem. C.

[23] Siemsen P., Livingston R. C., Diederich F. (2000). Acetylenic
coupling: A powerful tool
in molecular construction. Angew. Chem., Int.
Ed..

[24] Gao Y., Hou Y., Gámez F. G., Ferguson M. J., Casado J., Tykwinski R. R. (2020). The loss
of endgroup effects in long pyridyl-endcapped
oligoynes on the way to carbyne. Nat. Chem..

[25] Chalifoux W. A., Tykwinski R. R. (2009). Synthesis
of extended polyynes: Toward carbyne. C. R.
Chim..

[26] Chalifoux W. A., Tykwinski R. R. (2010). Synthesis of polyynes to model the sp-carbon allotrope
carbyne. Nat. Chem..

[27] Pigulski B., Gulia N., Szafert S. (2019). Reactivity
of Polyynes: Complex Molecules
from Simple Carbon Rods. Eur. J. Org. Chem..

[28] Pigulski B., Męcik P., Cichos J., Szafert S. (2017). Use of Stable Amine-Capped
Polyynes in the Regioselective Synthesis of Push – Pull Thiophenes. J. Org. Chem..

[29] Patrick C. W., Gao Y., Gupta P., Thompson A. L., Parker A. W., Anderson H. L. (2024). Masked
alkynes for synthesis of threaded carbon chains. Nat. Chem..

[30] Kudryavtsev, Y. U. P. Syntheses of Carbyne and Carbynoid Structures. In Physics and Chemistry of Materials with Low-Dimensional Structures; Springer, 1999; Vol. 21, pp 39–45.

[31] Cataldo, F. Polyynes: Synthesis, Properties, and Applications; CRC Press, 2005.

[32] Nishide D., Wakabayashi T., Sugai T., Kitaura R., Kataura H., Achiba Y., Shinohara H. (2007). Raman spectroscopy of size-selected
linear polyyne molecules C _2n_H_2_ (n = 4–6)
encapsulated in single-wall carbon nanotubes. J. Phys. Chem. C.

[33] Shi L., Rohringer P., Suenaga K., Niimi Y., Kotakoski J., Meyer J. C., Peterlik H., Wanko M., Cahangirov S., Rubio A. (2016). Confined linear carbon chains as a route to bulk carbyne. Nat. Mater..

[34] Lechner J. M. A., López P. H., Heeg S. (2022). Raman spectroscopy
of isolated carbyne chains confined in carbon nanotubes: Progress
and prospects. Chin. Phys. B.

[35] Feng Y., Zhang W., Tang K., Chen Y., Cao H., Hu Y., Cui W., Shi L., Yang G. (2025). Engineering external
and internal precursors to boost the synthesis of confined carbyne. Chem. Eng. J..

[36] Chen Y., Tang K., Zhang W., Cao H., Zhang H., Feng Y., Cui W., Hu Y., Shi L., Yang G. (2025). A Universal Method to Transform Aromatic Hydrocarbon
Molecules into
Confined Carbyne inside Single-Walled Carbon Nanotubes. ACS Nano.

[37] Okada S., Fujii M., Hayashi S. (2011). Immobilization of polyynes adsorbed
on Ag nanoparticle aggregates into poly­(vinyl alcohol) films. Carbon.

[38] Kim H., Tarakeshwar P., Fujikado N. M., Evraets K., Jones A. K., Meneghetti M., Buseck P. R., Sayres S. G. (2020). Pseudocarbynes:
Linear Carbon Chains Stabilized by Metal Clusters. J. Phys. Chem. C.

[39] An K., Wei G., Qi G., Sheng L., Yu L., Ren W., Zhao X. (2015). Stability improvement of C_8_H_2_ and C_10_H_2_ embedded in poly­(vinyl alcohol)
films with adsorption
on gold nanoparticles. Chem. Phys. Lett..

[40] Kucherik A. O., Osipov A. V., Arakelian S. M., Garnov S. V., Povolotckaya A. V., Kutrovskaya S. V. (2019). The laser-assisted
synthesis of linear carbon chains
stabilized by noble metal particle. J. Phys.:
Conf. Ser..

[41] Casari C. S., Russo V., Li Bassi A., Bottani C. E., Cataldo F., Lucotti A., Tommasini M., Del Zoppo M., Castiglioni C., Zerbi G. (2007). Stabilization of linear carbon structures
in a solid Ag nanoparticle assembly. Appl. Phys.
Lett..

[42] Matsutani R., Ozaki F., Yamamoto R., Sanada T., Okada Y., Kojima K. (2009). Preparation of polyynes up to C_22_H_2_ by liquid-phase laser ablation and their immobilization into SiO_2_ gel. Carbon.

[43] Sata R., Suzuki H., Ueno N., Morisawa Y., Hatanaka M., Wakabayashia T. (2019). UV-polarizing
linear polyyne molecules aligned in PVA. Chin.
J. Chem. Phys..

[44] Peggiani S., Facibeni A., Milani A., Castiglioni C., Russo V., Li Bassi A., Casari C. S. (2020). In situ synthesis
of polyynes in a polymer matrix via pulsed laser ablation in a liquid. Mater. Adv..

[45] Wendorff, J. H. ; Agarwal, S. ; Greiner, A. Electrospinning: Materials, Processing, and Applications; John Wiley & Sons, 2012.

[46] Bhardwaj N., Kundu S. C. (2010). Electrospinning: A fascinating fiber fabrication technique. Biotechnol. Adv..

[47] Teo W. E., Ramakrishna S. (2006). A review on
electrospinning design and nanofibre assemblies. Nanotechnology.

[48] Aflaha R., Putri L. A., Farrel A., Anzinger S., Rianjanu A., Yulianto N., Fueldner M., Roto R., Peiner E., Wasisto H. S., Triyana K. (2025). Crafting high-temperature
stable
and hydrophobic nanofiber membranes for particulate matter filtration. Commun. Mater..

[49] Angammana C. J., Jayaram S. H. (2016). Fundamentals of electrospinning and
processing technologies. Part. Sci. Technol..

[50] Ahmadian A., Shafiee A., Aliahmad N., Agarwal M. (2021). Overview of nano-fiber
mats fabrication via electrospinning and morphology analysis. Textiles.

[51] Jung J. W., Lee C. L., Yu S., Kim I. D. (2016). Electrospun nanofibers
as a platform for advanced secondary batteries: A comprehensive review. J. Mater. Chem. A.

[52] Hu H. Y., Xie N., Wang C., Wu F., Pan M., Li H. F., Wu P., Wang X. Di., Zeng Z., Deng S. (2019). Enhancing
the performance of motive power lead-acid batteries by high surface
area carbon black additives. Appl. Sci..

[53] Blackman J. M., Patrick J. W., Arenillas A., Shi W., Snape C. E. (2006). Activation
of carbon nanofibres for hydrogen storage. Carbon.

[54] Im J. S., Park S. J., Kim T. J., Kim Y. H., Lee Y. S. (2008). The study
of controlling pore size on electrospun carbon nanofibers for hydrogen
adsorption. J. Colloid Interface Sci..

[55] Chen L. F., Zhang X. D., Liang H. W., Kong M., Guan Q. F., Chen P., Wu Z. Y., Yu S. H. (2012). Synthesis of nitrogen-doped
porous carbon nanofibers as an efficient electrode material for supercapacitors. ACS Nano.

[56] Teo E. Y. L., Muniandy L., Ng E. P., Adam F., Mohamed A. R., Jose R., Chong K. F. (2016). High surface area activated carbon
from rice husk as a high performance supercapacitor electrode. Electrochim. Acta.

[57] Chen S., Xing W., Duan J., Hu X., Qiao S. Z. (2013). Nanostructured
morphology control for efficient supercapacitor electrodes. J. Mater. Chem. A.

[58] Beilke M. C., Zewe J. W., Clark J. E., Olesik S. V. (2013). Aligned electrospun
nanofibers for ultra-thin layer chromatography. Anal. Chim. Acta.

[59] Bhattarai N., Edmondson D., Veiseh O., Matsen F. A., Zhang M. (2005). Electrospun
chitosan-based nanofibers and their cellular compatibility. Biomaterials.

[60] Yu L., Zhongbiao S., Xu L., Mingdi W. (2017). High Throughput Preparation
of Aligned Nanofibers. Polymers.

[61] Kiselev P., Rosell-llompart J. (2012). Highly Aligned Electrospun Nanofibers by Elimination
of the Whipping Motion. J. Appl. Polym. Sci..

[62] Haider S., Al-zeghayer Y., Ali F. A. A., Imran M., Aijaz M. O. (2013). Highly
aligned narrow diameter chitosan electrospun nanofibers. J. Polym. Res..

[63] Omer M., Rezaul M., Alharbi H. F., Alharthi N. H. (2019). Novel optimized
highly aligned electrospun PEI-PAN nanofibre mats with excellent wettability. Polymer.

[64] Mariappan V. K., Krishnamoorthy K., Manoharan S., Pazhamalai P., Kim S. J. (2021). Electrospun Polymer-Derived
Carbyne Supercapacitor
for Alternating Current Line Filtering. Small.

[65] Pigulski B., Gulia N., Mȩcik P., Wieczorek R., Arendt A., Szafert S. (2019). Crystal Engineering
of 1-Halopolyynes
by End-Group Manipulation. Cryst. Growth Des..

[66] Gulia N., Pigulski B., Charewicz M., Szafert S. (2014). A Versatile and Highly
Efficient Method for 1-Chlorination of Terminal and Trialkylsilyl-Protected
Alkynes. Chem. - Eur. J..

[67] Wada Y., Morisawa Y., Wakabayashi T. (2012). Spectroscopic characterization of
a series of polyyne – iodine molecular complexes H­(CC)_n_H­(I_6_) of n = 5 – 9. Chem. Phys. Lett..

[68] East A. L. L., Grittner K. L., Afzal A. I., Simpson A. G., Liebman J. F. (2005). Length
and Substituent-Scrambling Energies of Parent and Halogen-Substituted
Conjugated Polyynes. J. Phys. Chem. A.

[69] Melesi S., Marabotti P., Milani A., Pigulski B., Gulia N., Pińkowski P., Szafert S., Del Zoppo M., Castiglioni C., Casari C. S. (2024). Impact of Halogen Termination and
Chain Length on π-Electron Conjugation and Vibrational Properties
of Halogen-Terminated Polyynes. J. Phys. Chem.
A.

[70] Milani A., Tommasini M., Zerbi G. (2009). Connection among Raman wavenumbers,
bond length alternation and energy gap in polyynes. J. Raman Spectrosc..

[71] Ku̅rti J., Magyar C., Balázs A., Rajczy P. (1995). Vibrational analysis
for short carbon chains with alternating and cumulenic structure. Synth. Met..

[72] Heimann, R. B. ; Evsyukov, S. E. ; Kavan, L. Carbyne and Carbynoid Structures; Springer Science & Business Media, 1999.

[73] Dirlikov S., Koenig J. L. (1980). Carbon–hydrogen stretching and bending vibrations
in the Raman spectra of poly (methylmethacrylate). J. Raman Spectrosc..

[74] Nikitin V. N., Mikhailova N. V., Volkova L. A. (1965). Crystallization of stereoregular
polydimethylmethacrylate. Polym. Sci. U.S.S.R..

[75] Marabotti P., Milani A., Lucotti A., Brambilla L., Tommasini M., Casari C. S. (2021). Vibrational
and nonlinear
optical properties of amine-capped push-pull polyynes by infrared
and Raman spectroscopy. Carbon Trends.

[76] Zha B., Li J., Wu J., Miao X., Zhang M. (2019). Cooperation and competition
of hydrogen and halogen bonds in 2D self-assembled nanostructures
based on bromine substituted coumarins. New
J. Chem..

[77] Zha B., Dong M., Miao X., Miao K., Hu Y., Wu Y., Xu L., Deng W. (2016). Controllable Orientation of Ester-Group-Induced
Intermolecular Halogen Bonding in a 2D Self-Assembly. J. Phys. Chem. Lett..

[78] Kampes R., Zechel S., Hager M. D., Schubert U. S. (2021). Halogen
bonding
in polymer science: Towards new smart materials. Chem. Sci..

[79] Arrigoni A., Serra G., Manidi J., Bertarelli C., Castiglioni C. (2022). Morphology and Intramolecular Interactions
in P­(VDF-TrFE)
Electrospun Nanofibers Doped with Disperse Orange 3 Dye: A Joint Infrared
Spectroscopy and Electron Microscopy Study. ACS Omega.

[80] Li Q. Y., Yao Z. F., Wang J. Y., Pei J. (2021). Multi-level aggregation
of conjugated small molecules and polymers: From morphology control
to physical insights. Rep. Prog. Phys..

[81] Teng H., Koike K., Zhou D., Satoh Z., Koike Y., Okamoto Y. (2009). High glass transition
temperatures of poly­(methyl methacrylate)
prepared by free radical initiators. J. Polym.
Sci., Part A:Polym. Chem..

[82] Cavallo G., Metrangolo P., Milani R., Pilati T., Priimagi A., Resnati G., Terraneo G. (2016). The halogen bond. Chem. Rev..

[83] Aflaha R., Maharani C. N., Prabowo Y. D., Roto R., Gupta R., Wasisto H. S., Rianjanu A., Putri W. B. K., Triyana K. (2025). Environmentally
friendly waterproof and breathable nanofiber membranes with ultra-low
pressure drop and high performance. Colloids
Surf., A.

[84] Wagner P. J., Sedon J. H., Gudmundsdottir A. (1996). Photoinduced Radical Cleavage of
Bromophenyl Ketones. J. Am. Chem. Soc..

[85] Nanjundiah B. S., Sonawane H. R. (1985). Photochemistry of
the carbon-halogen bond: some recent
developments. Proc. Indian Acad. Sci..

[86] Raviola C., Fagnoni M. (2018). Search for a photoinduced
(site-selective) cleavage
of the Ar-Cl bond in dichloroanisoles. Photochem.
Photobiol. Sci..

[87] Patterson G. H., Piston D. W. (2000). Photobleaching in two-photon excitation microscopy. Biophys. J..

[88] Piedra H. F., Valdés C., Plaza M. (2023). Shining light on halogen-bonding
complexes: a catalyst-free activation mode of carbon-halogen bonds
for the generation of carbon-centered radicals. Chem. Sci..

