# Supplementary material for: Probing the Stability of Halogenated Carbon Atomic Wires in Electrospun Nanofibers via Raman Spectroscopy
Source: J Phys Chem C Nanomater Interfaces. 2025 Jul 8;129(28):12916–26. doi: 10.1021/acs.jpcc.5c02960 (PMC12278307; doi:10.1021/acs.jpcc.5c02960)
Supplement: Supplementary file 1 [file jp5c02960_si_001.pdf]

Supporting information for

“Probing the Stability of Halogenated Carbon Atomic Wires in Electrospun Nanofibers via Raman Spectroscopy”

*Simone Melesi [a], Piotr Pińkowski [b], Bartłomiej Pigulski [b], Nurbey Gulia [b], Sławomir Szafert [b], Chiara Bertarelli [c], Chiara Castiglioni [c], Carlo S. Casari [a]**

a Department of Energy, Micro and Nanostructured Materials Laboratory - NanoLab, Energy, Politecnico di Milano, Via Ponzio 34/3, Milano 20133, Italy
b Faculty of Chemistry, University of Wrocław, 14 F. Joliot-Curie, Wrocław 50-383, Poland
c Department of Chemistry, Materials and Chemical Engineering “Giulio Natta”, Politecnico di Milano Piazza Leonardo da Vinci 32, Milano 20133, Italy

Table of contents:

1. Effect of the electrospinning parameters and SEM/EDX analysis S2
2. Raman investigation of the homogeneity of CAWs distribution S7
3. Raman investigation of nanocomposites’ stability S8
   a. Time stability S8
   b. Thermal stability S11
   c. Photodegradation stability S15
4. References S19

1. Effect of the electrospinning parameters and SEM/EDX analysis

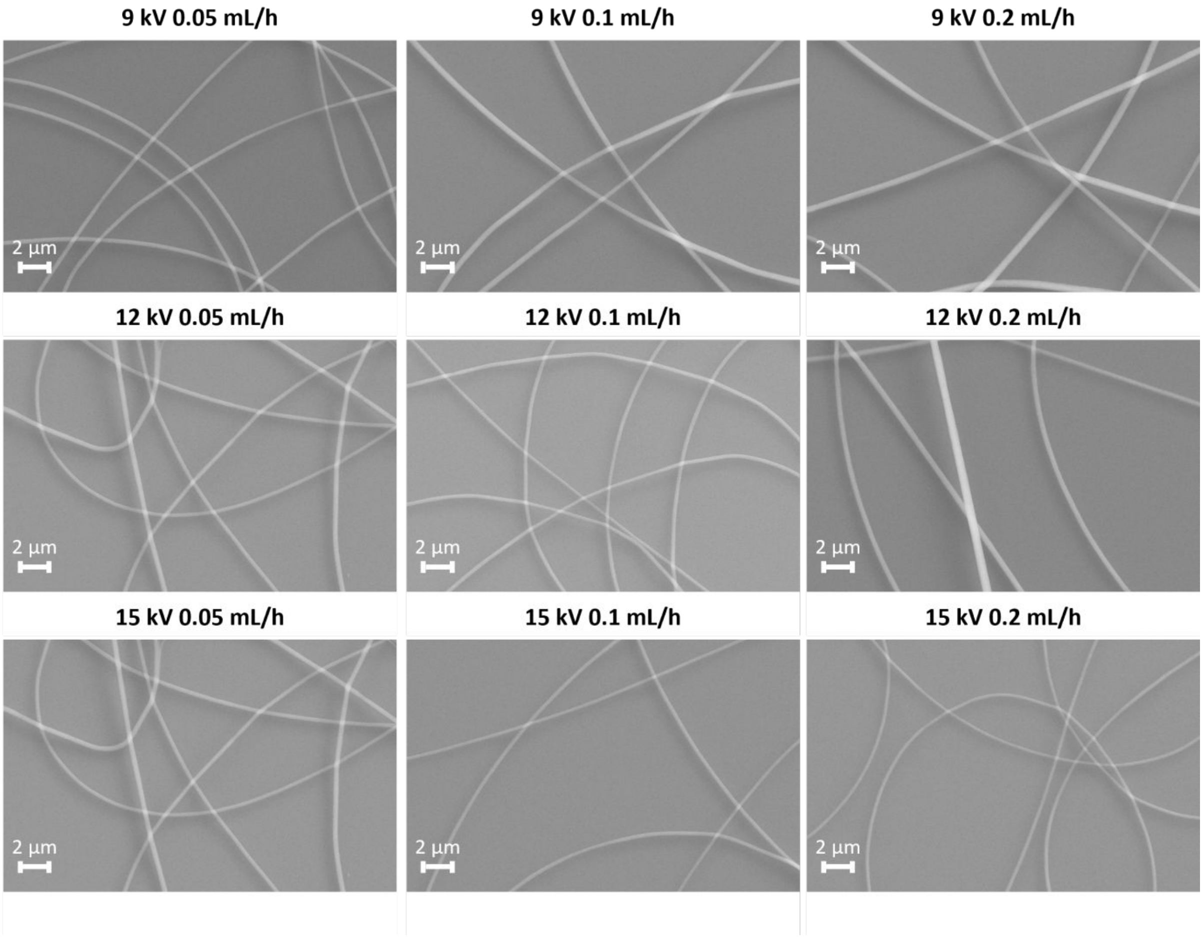

Fig. S.1 SEM images of the PMMA nanofibers electrospun from a PMMA (6 wt%) solution in DMF. Different images represent nanofibers obtained at a needle-to-collector distance D = 15 cm and with different applied voltages (V = 9, 12, 15 kV) and flow rates (ϕ = 0.05, 0.1, 0.2 mL/h).

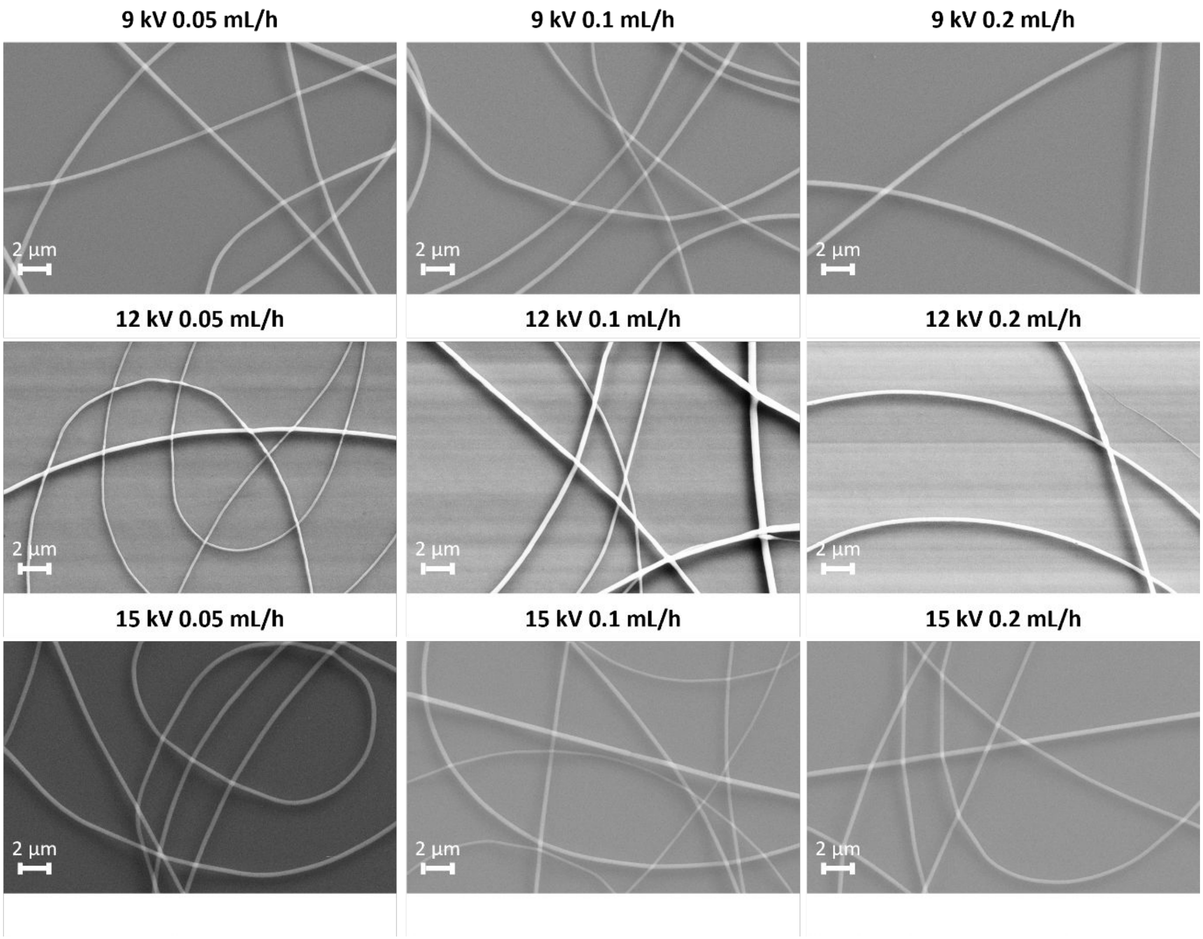

Fig. S.2 SEM images of the PMMA nanofibers electrospun from a PMMA (6 wt%) solution in DMF. Different images represent nanofibers obtained at a needle-to-collector distance D = 20 cm and with different applied voltages (V = 9, 12, 15 kV) and flow rates (ϕ = 0.05, 0.1, 0.2 mL/h).

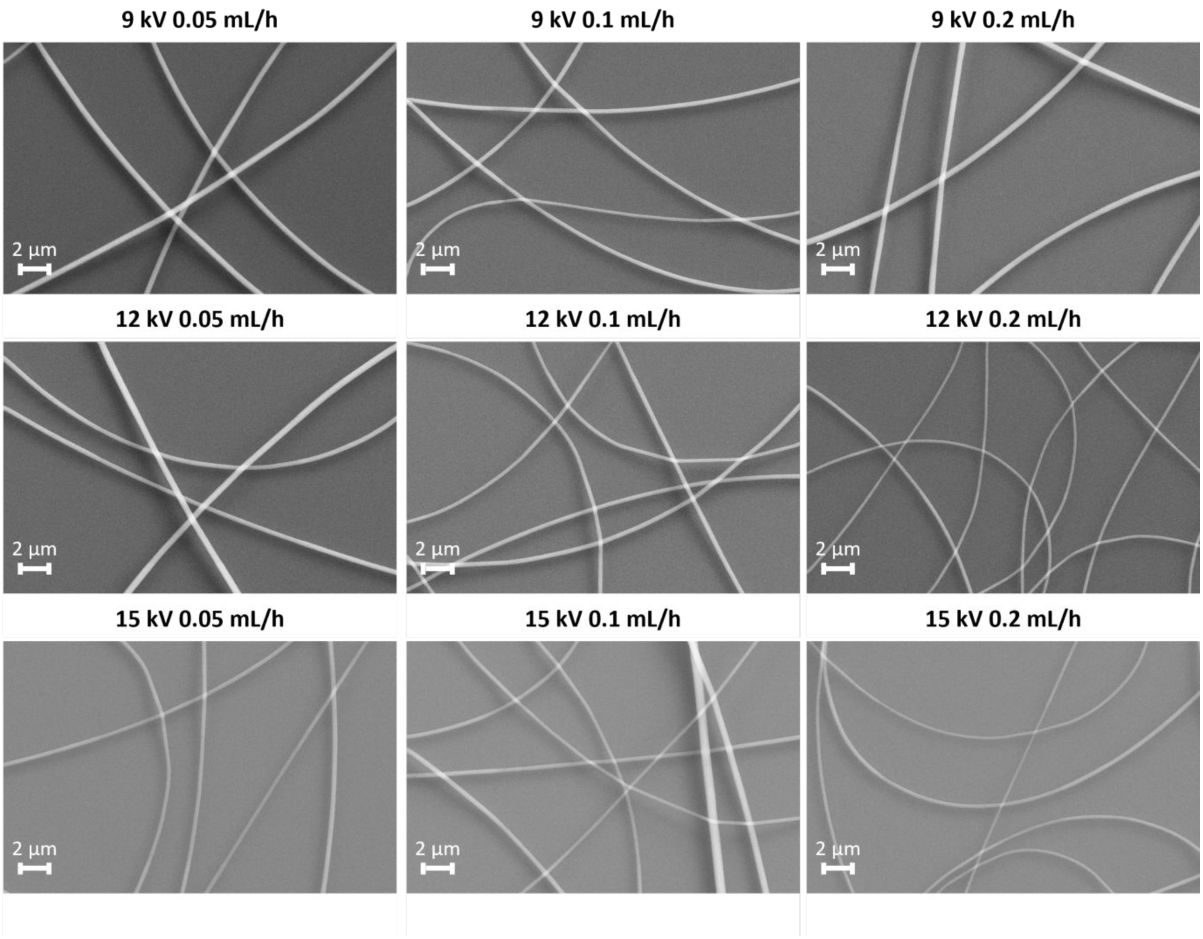

Fig. S.3 SEM images of the PMMA nanofibers electrospun from a PMMA (6 wt%) solution in DMF. Different images represent nanofibers obtained at a needle-to-collector distance D = 25 cm and with different applied voltages (V = 9, 12, 15 kV) and flow rates (ϕ = 0.05, 0.1, 0.2 mL/h).

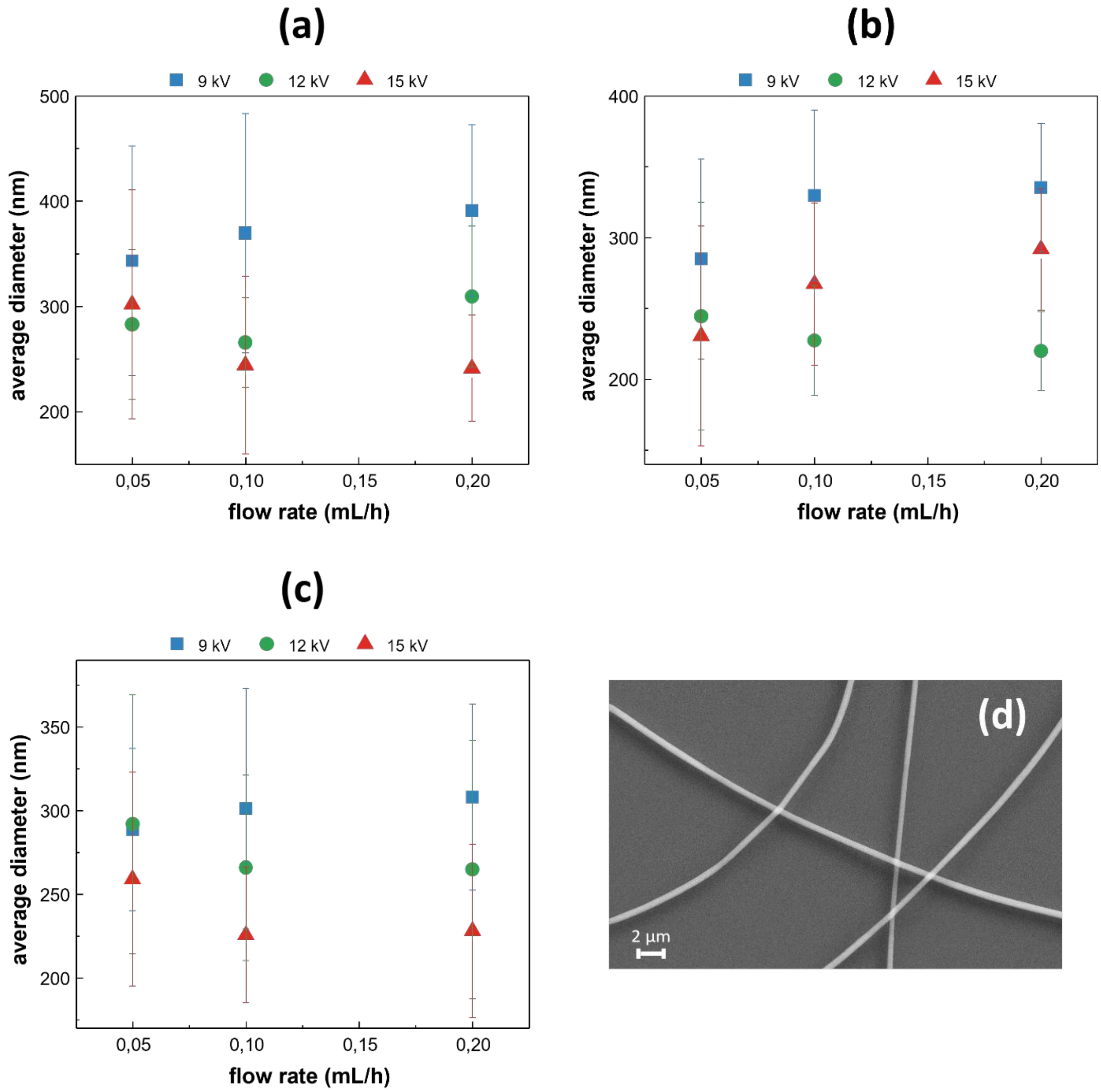

Fig. S. 4 Average diameters for all the PMMA nanofibers collected at different electrospinning parameters. In a), b), and c) the average diameters obtained at D = 15, 20, and 25 cm, respectively. In d) a SEM image of the nanofibers obtained with the optimized parameters (i.e., V = 12kV, D = 20 cm, ϕ = 0.2 mL/h). These are the conditions that lead to the lower average diameter and standard deviation.

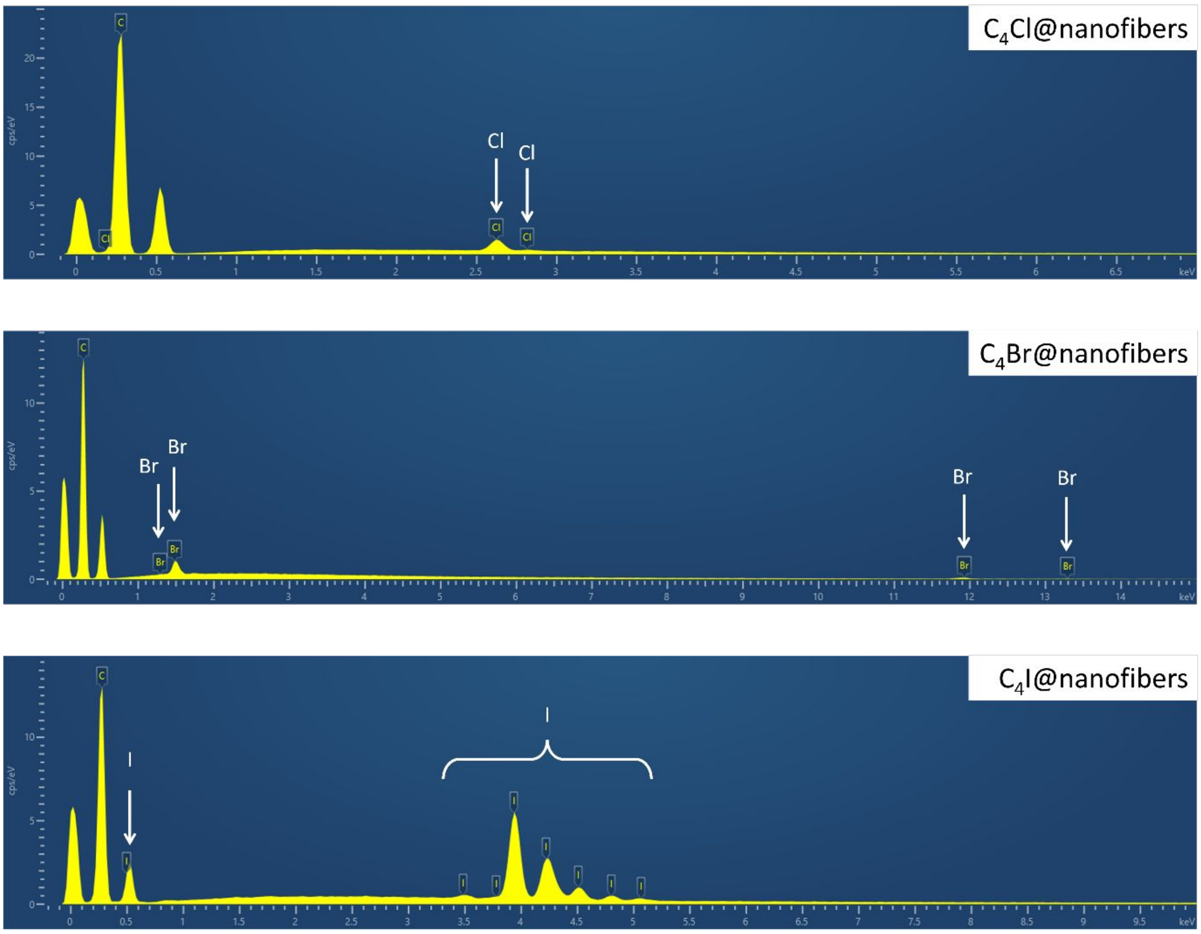

Fig. S. 5 EDX spectra of electrospun PMMA nanofibers embedding C_4Cl, C_4Br, and C_4I, respectively. The halogen atom peaks are highlighted.

2. Raman investigation of the homogeneity of CAWs distribution

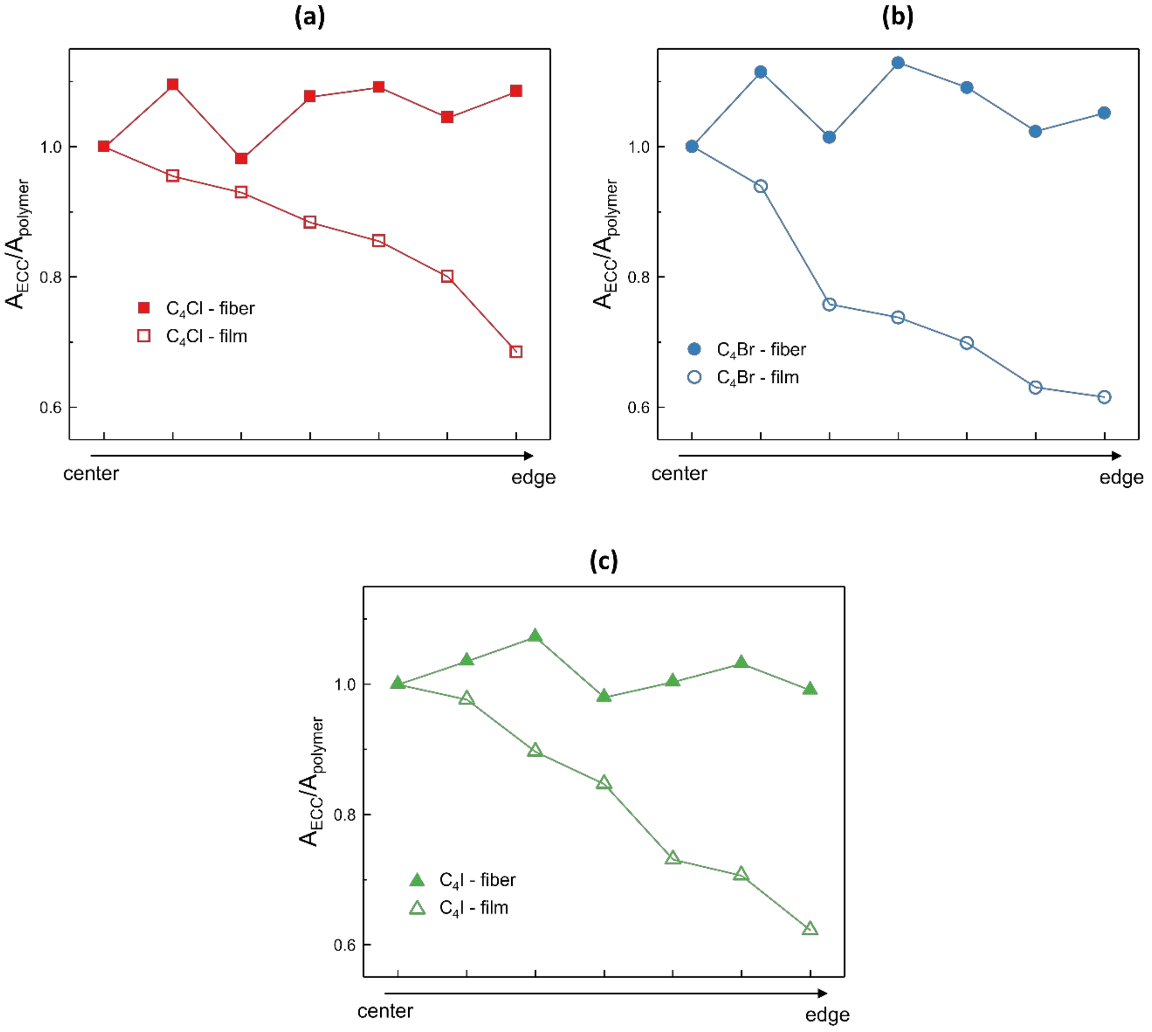

(d)

	% variation from different spot (A_{ECC}/A_{PMMA})	
	**Nanofibers membrane**	**Drop-casted film**
**PMMA + C_4Cl**	4.8	10.2
**PMMA + C_4Br**	5.5	13.9
**PMMA + C_4I**	3.6	17.3

Fig. S. 6 Ratio between the areas of the ECC band and the PMMA CH-stretching band for electrospun membranes (solid symbols) and drop-casted films (hollow symbols), measured at different positions across the samples, from the center to the edges. In (a), (b), and (c) the results for C_4Cl, C_4Br, and C_4I, respectively. In table (d), the percentage variation of the ratio between the area of the CAW ECC Raman band and the area of the CH stretching band of PMMA is reported for all the samples, both in the form of electrospun (nanofibers) membranes and drop-casted films.

3. Raman investigation of nanocomposites' stability

a. Time stability

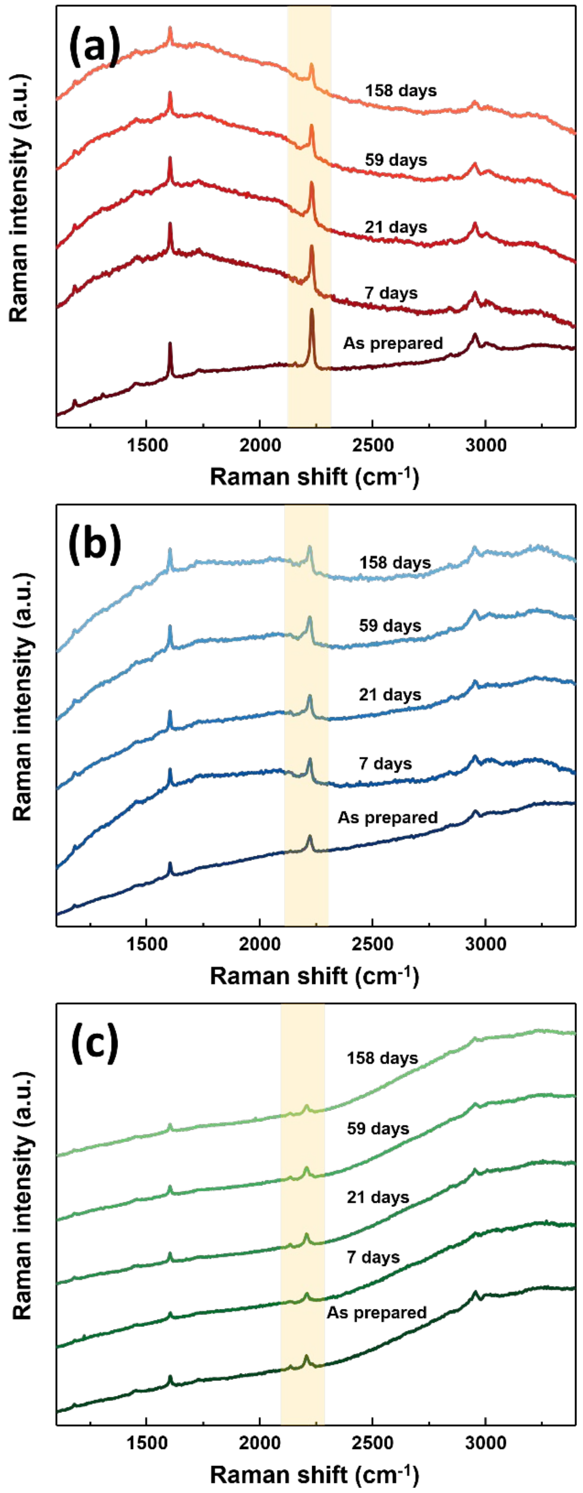

Fig. S. 7 Raman spectra of the electrospun PMMA membranes containing C_4X halogenated CAW (X = Cl, Br, I) measured at different times after electrospinning. In (a), (b), and (c) spectra of the C_4Cl, C_4Br, and C_4I fibers respectively. Highlighted in yellow is the CAW ECC region.

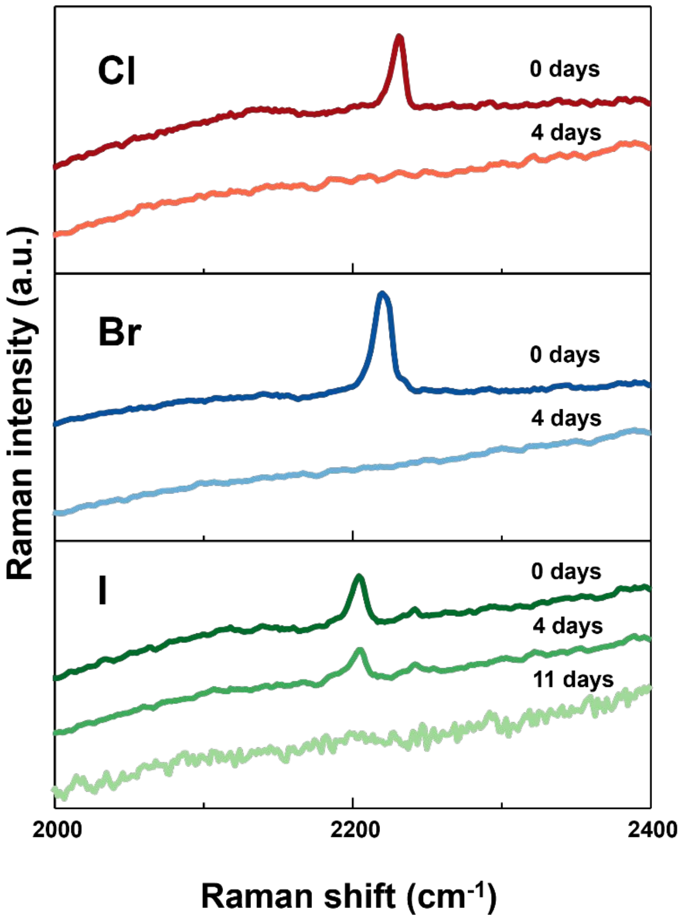

Fig. S. 8 Raman spectra of the C_4X halogenated CAW (X = Cl, Br, I) in the form of powders and measured at different times.

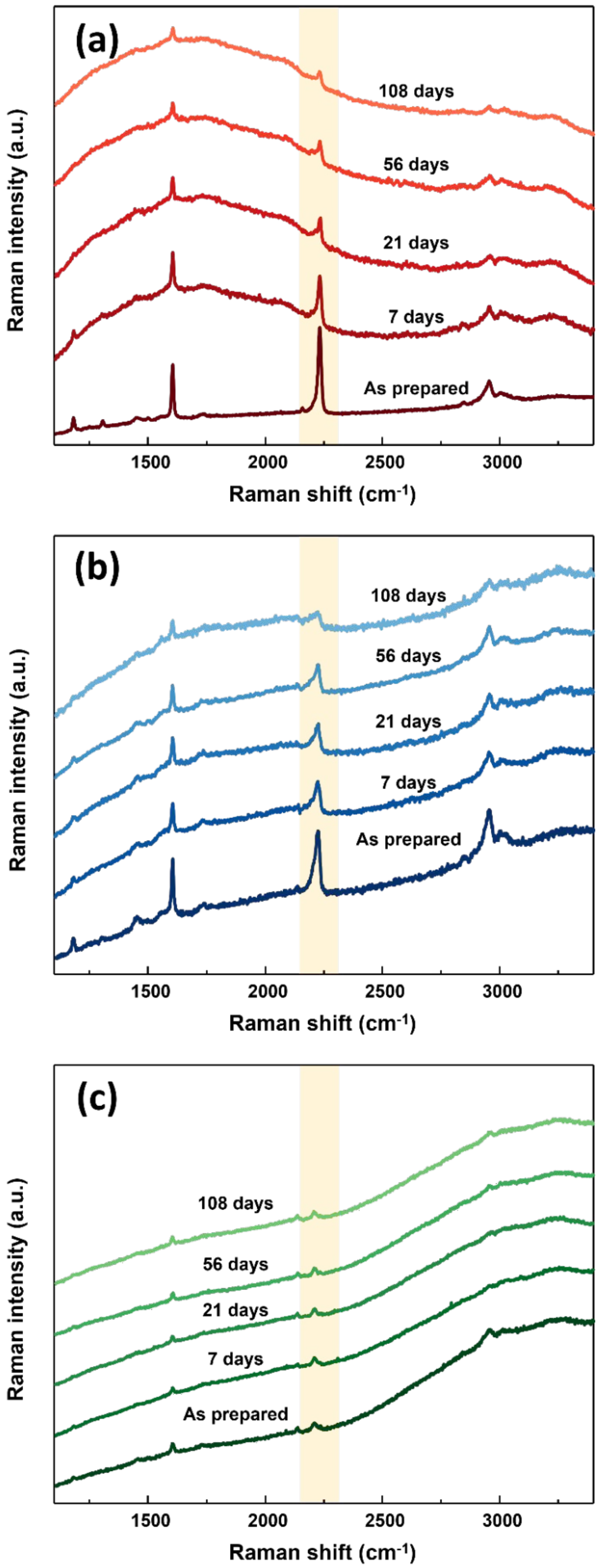

Fig. S. 9 Raman spectra of the drop casted PMMA thin films containing C_4X halogenated CAW (X = Cl, Br, I) measured at different times after deposition. In (a), (b), and (c) spectra of the C_4Cl, C_4Br, and C_4I films respectively. Highlighted in yellow is the CAW ECC region.

b. Thermal stability

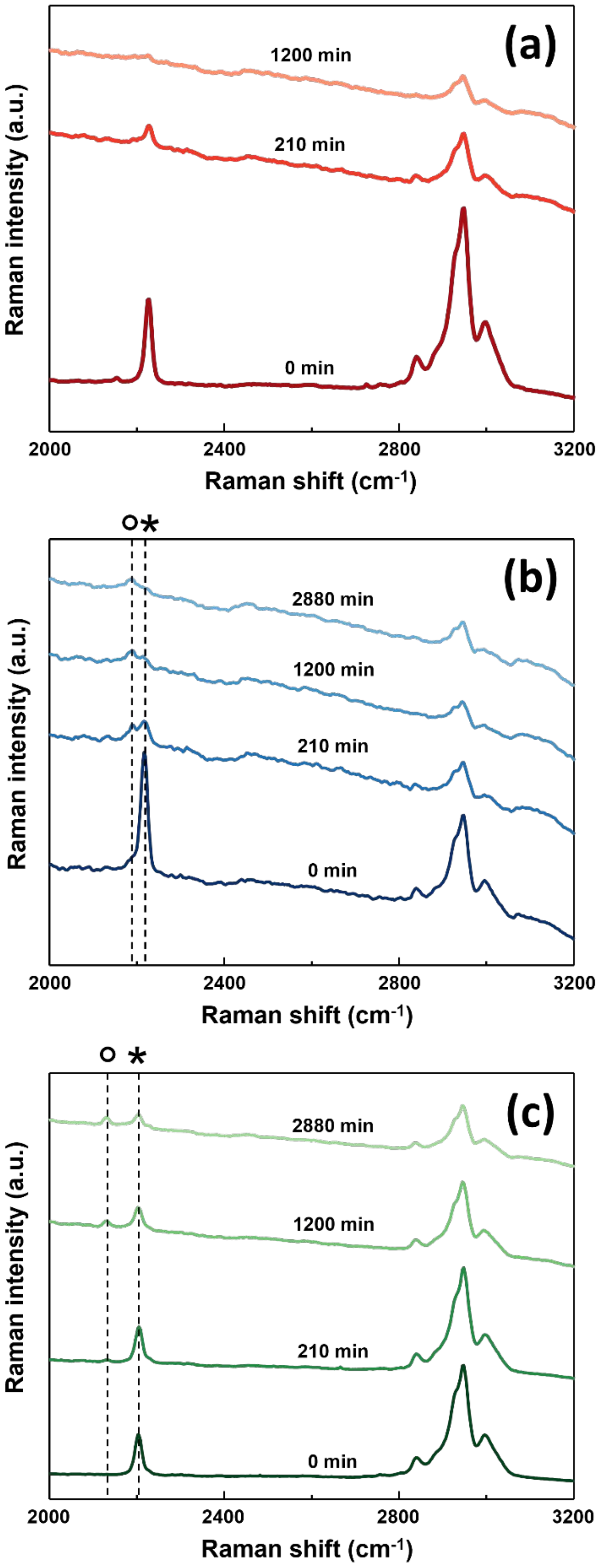

*Fig. S. 10 Raman spectra of the electrospun PMMA membranes containing C_4X halogenated CAW(X = Cl, Br, I) measured after heating at 90 °C for different times. In (a), (b), and (c) spectra of the C_4Cl, C_4Br, and C_4I fibers respectively. In (b) and (c) the dashed lines represent the peaks frequencies of the CAW ECC (indicated with *) and the secondary peaks (indicated with °).*

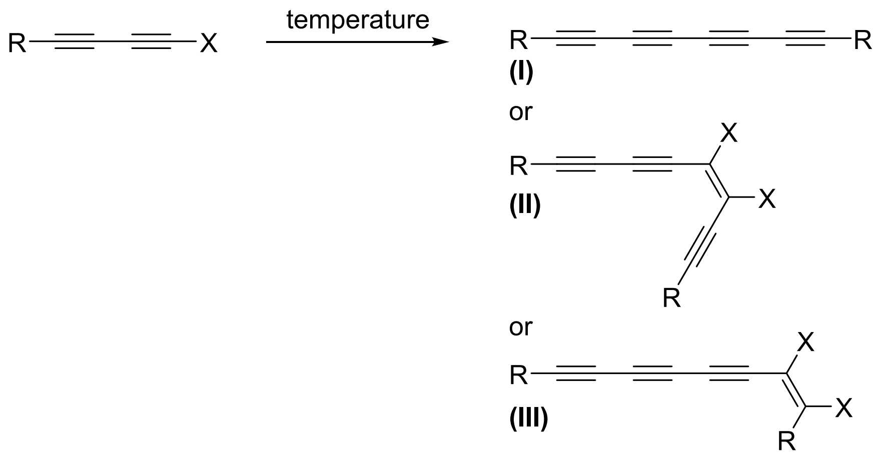

Fig. S. 11 Possible degradation pathways of halogenated CAWs.

Based on the known reactivity of 1-haloalkynes, possible degradation pathways for halogenated CAWs can be proposed (Fig S.11). One such pathway is homocoupling, which leads to a doubling of the carbon chain length (structure **I**); this is a well-known process that can occur at elevated temperatures.[1] Dimerization resulting in enyne formation has also been reported, [2] and the formation of structures **II** and **III** may be considered in this context.[2]

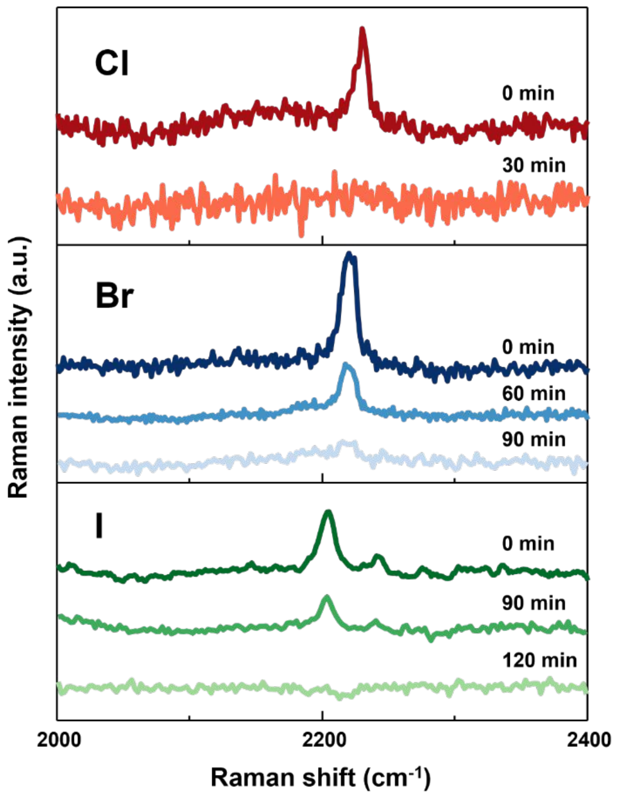

Fig. S. 12 Raman spectra of the C_4X halogenated CAW (X = Cl, Br, I) in the form of powders measured after heating at 90 °C for different times.

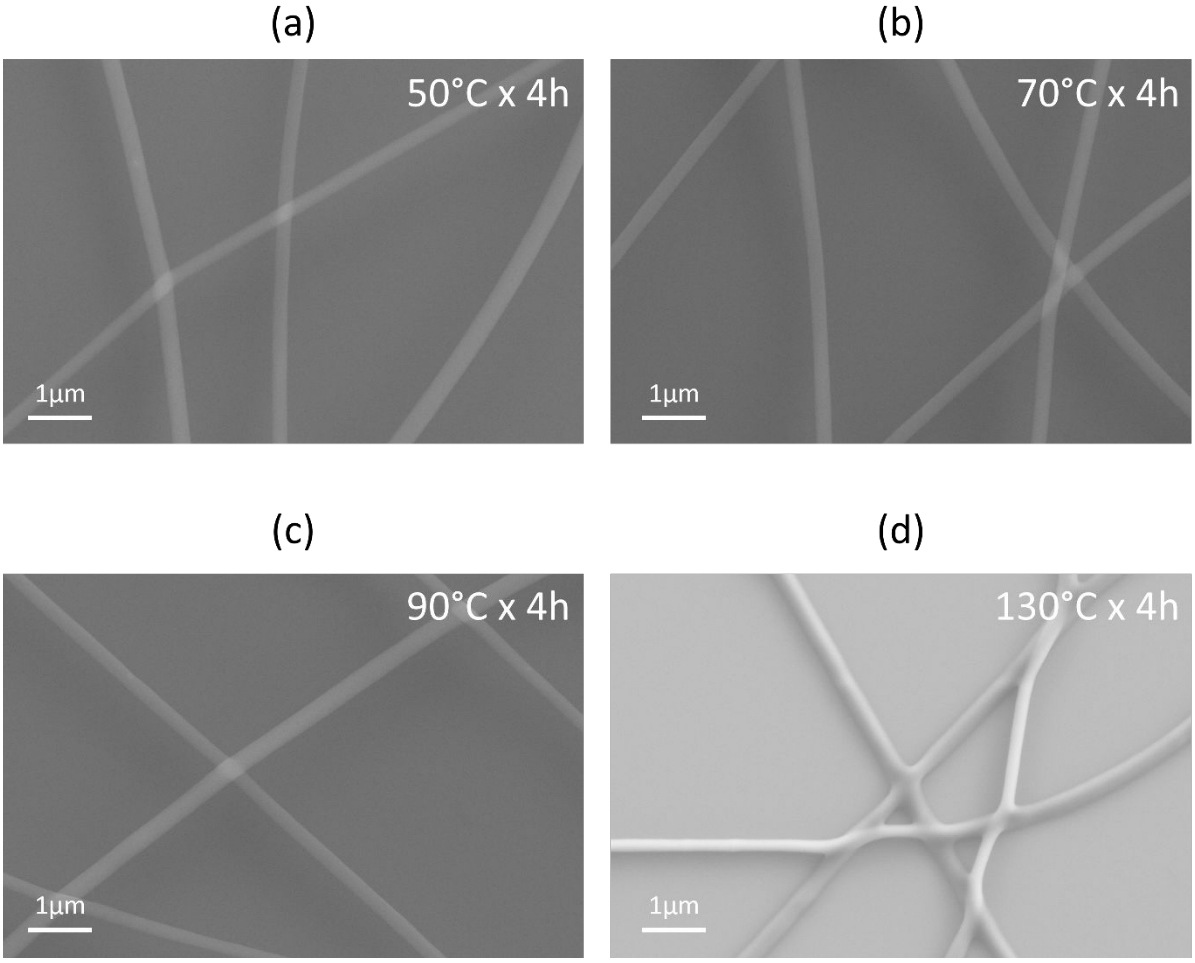

Fig. S. 13 SEM images of PMMA electrospun nanofibers after exposure at different temperatures for 4 hours. In (a), (b), (c), and (d) images of nanofibers exposed to 50, 70, 90, and 130 °C, respectively.

c. Photodegradation stability

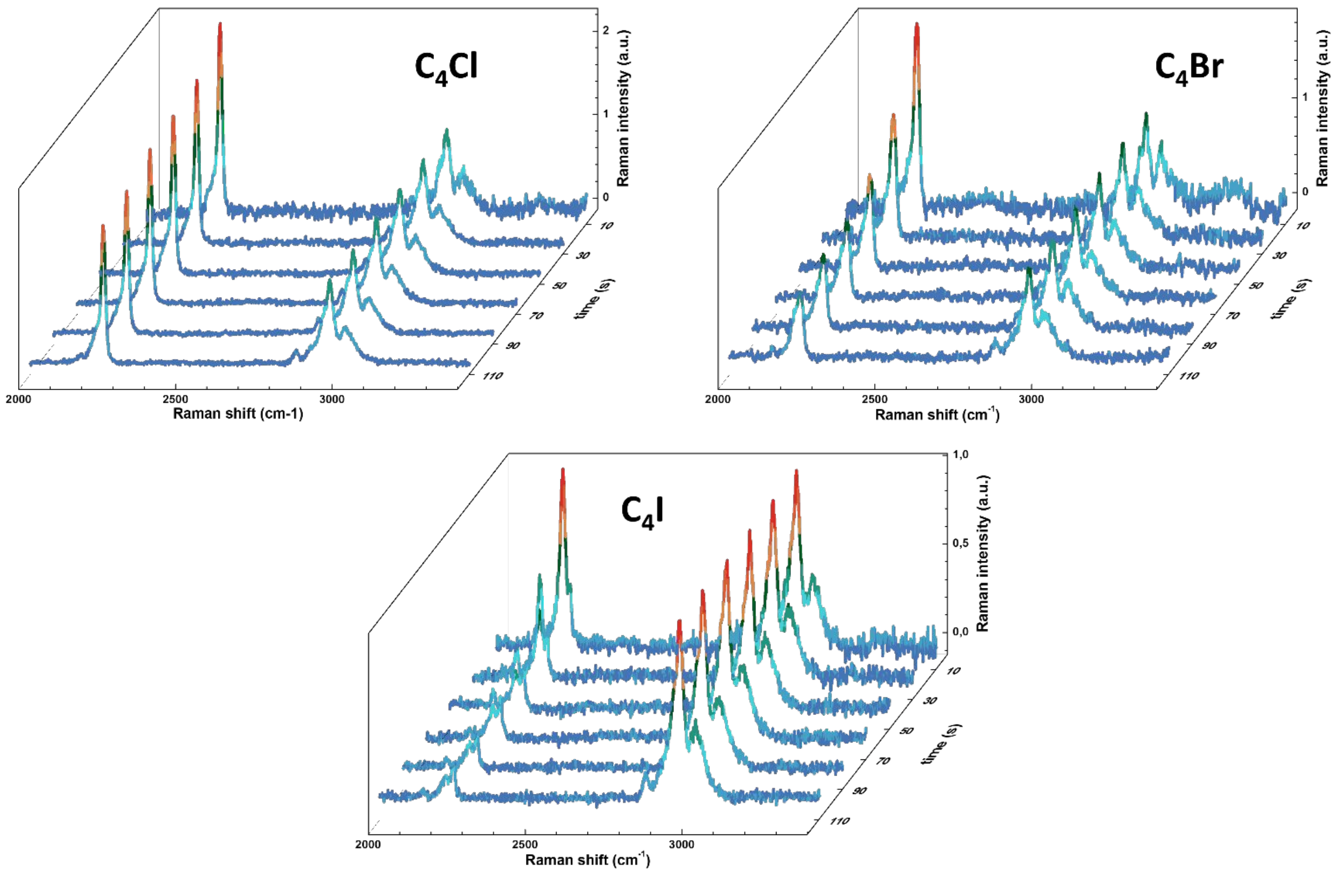

Fig. S. 14 Raman spectra of the electrospun PMMA membranes containing the C_4X halogenated CAW (X = Cl, Br, I) measured at different times of exposure to light irradiation (532 nm).

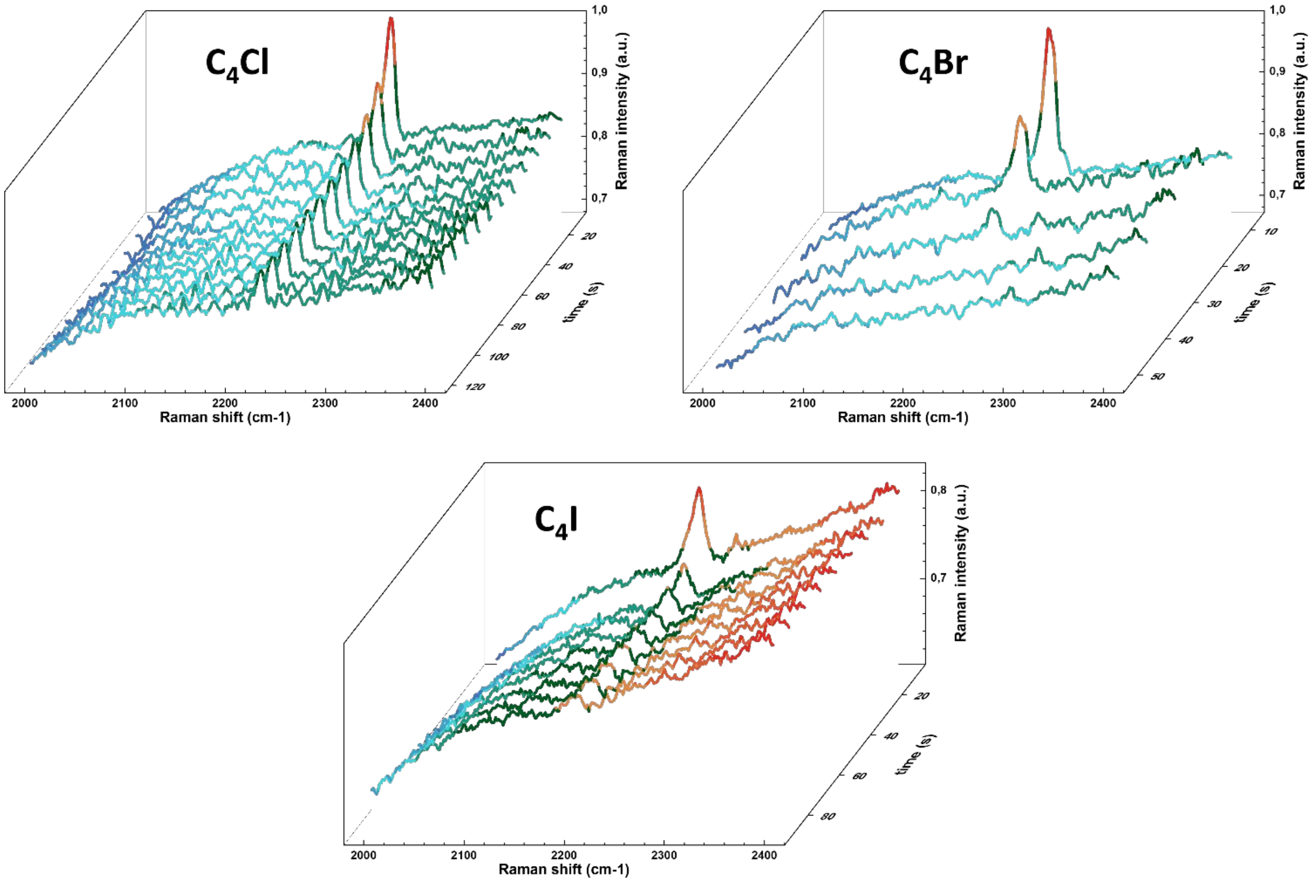

Fig. S. 15 Raman spectra of the C_4X halogenated CAW (X = Cl, Br, I) in the form of powders and measured at different times of exposure to light irradiation (532 nm).

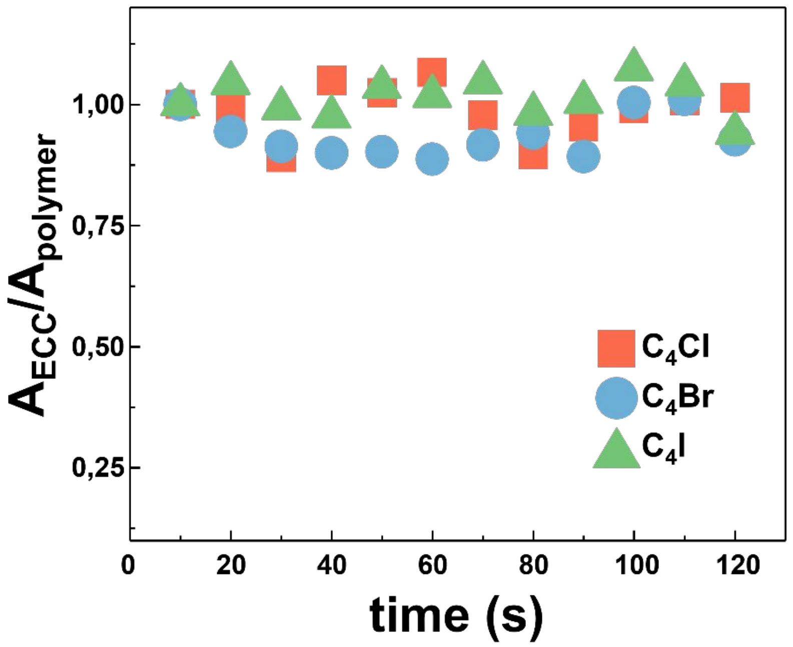

Fig. S. 16 Evolution of the ECC band areas for the electrospun membranes of the C_4X halogenated CAW (X = Cl, Br, I) after exposure to light irradiation (660 nm) for different times. The ratio between the areas of the CAW ECC band and the PMMA CH-stretching band is considered.

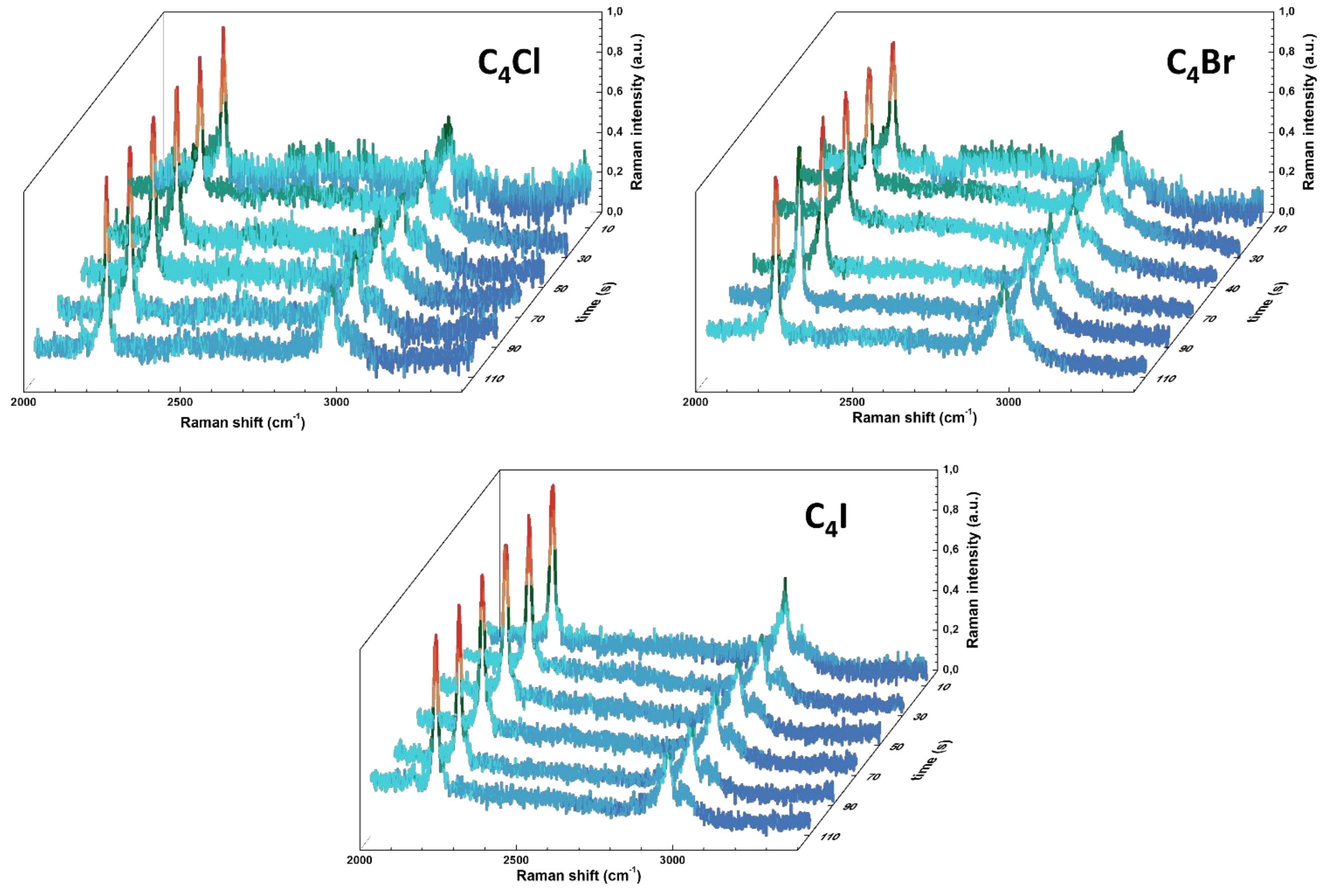

Fig. S. 17 Raman spectra of the electrospun PMMA membranes containing the C_4X halogenated CAW (X = Cl, Br, I) measured at different times of exposure to light irradiation (660 nm).

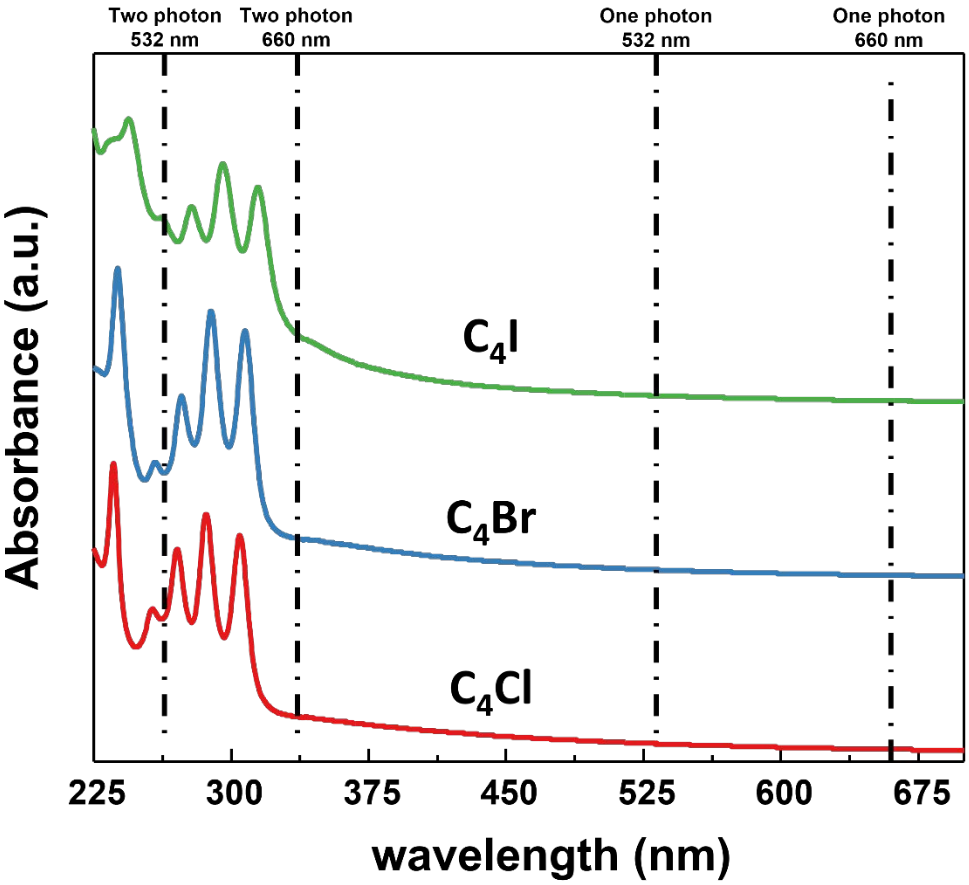

Fig. S. 18 UV-Vis spectra of the C_4X halogenated CAW (X = Cl, Br, I) dissolved in acetonitrile with a concentration of 10^{-5} mol/L. With the dashed lines the excitation wavelengths of the two lasers (532 nm and 660 nm) are represented together along with their relative two-photon combinations.

4. References

1) Chen, Z.; Jiang, H.; Wang, A.; Yang, S. Transition-Metal-Free Homocoupling of 1-Haloalkynes: A Facile Synthesis of Symmetrical 1,3-Diynes. *J. of Org. Chem.* **2010**, *75* (19), 6700–6703. https://doi.org/10.1021/jo101216m.

2) Hoheisel, T. N.; Schrettl, S.; Marty, R.; Todorova, T. K.; Corminboeuf, C.; Sienkiewicz, A.; Scopelliti, R.; Schweizer, W. B.; Frauenrath, H. A Multistep Single-Crystal-to-Single-Crystal Bromodiacetylene Dimerization. *Nat. Chem.* **2013**, *5* (4), 327–334. https://doi.org/10.1038/nchem.1575.
